# ERRα mediates metabolic adaptations driving lapatinib resistance in breast cancer

**DOI:** 10.1038/ncomms12156

**Published:** 2016-07-12

**Authors:** Geneviève Deblois, Harvey W. Smith, Ingrid S. Tam, Simon-Pierre Gravel, Maxime Caron, Paul Savage, David P. Labbé, Louis R. Bégin, Michel L. Tremblay, Morag Park, Guillaume Bourque, Julie St-Pierre, William J. Muller, Vincent Giguère

**Affiliations:** 1 Goodman Cancer Research Centre, McGill University, Montréal, Québec, Canada H3A 1A3; 2 Department of Biochemistry, McGill University, Montréal, Québec, Canada H3G 1Y6; 3 Department of Human Genetics, McGill University, Montréal, Québec, Canada H3G 1Y6; 4 Department of Medicine, McGill University, Montréal, Québec, Canada H3A 1A3; 5 Service d'anatomopathologie, Hôpital du Sacré-Cœur de Montréal, 5400 Boulevard Gouin Ouest, Montréal, Québec, Canada H4J 1C5; 6 Department of Oncology, McGill University, Montréal, Québec, Canada H2W 1S6

## Abstract

Despite the initial benefits of treating HER2-amplified breast cancer patients with the tyrosine kinase inhibitor lapatinib, resistance inevitably develops. Here we report that lapatinib induces the degradation of the nuclear receptor ERRα, a master regulator of cellular metabolism, and that the expression of ERRα is restored in lapatinib-resistant breast cancer cells through reactivation of mTOR signalling. Re-expression of ERRα in resistant cells triggers metabolic adaptations favouring mitochondrial energy metabolism through increased glutamine metabolism, as well as ROS detoxification required for cell survival under therapeutic stress conditions. An ERRα inverse agonist counteracts these metabolic adaptations and overcomes lapatinib resistance in a HER2-induced mammary tumour mouse model. This work reveals a molecular mechanism by which ERRα-induced metabolic reprogramming promotes survival of lapatinib-resistant cancer cells and demonstrates the potential of ERRα inhibition as an effective adjuvant therapy in poor outcome HER2-positive breast cancer.

## Abstract (web summary)

Despite initial benefits in treating HER2-positive breast cancer patients with lapatinib, resistance is prevalent. Here the authors show that lapatinib resistance can be ascribed to mTOR-mediated re-activation of ERRα and to the consequent induction of a metabolic adaptation.

## Graphical abstract

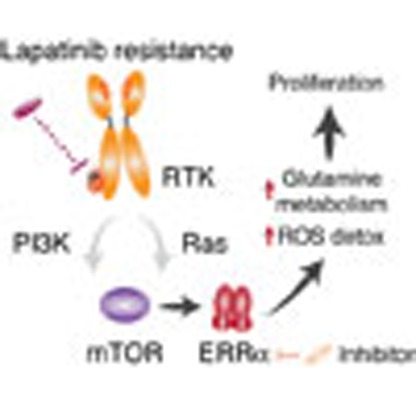

The overexpression and aberrant activation of receptor tyrosine kinase (RTK) signalling pathways constitute a prominent driver of human breast cancer progression^1^,^2^. Lapatinib (Tykerb) is a dual epidermal growth factor receptor (EGFR)/human EGFR-2 (HER2) tyrosine kinase inhibitor (TKI) approved for patients with HER2-amplified breast tumours presenting with metastatic lesions^3^. Despite initial benefits of lapatinib treatment in breast cancer patients, resistance inevitably develops^4^. While several studies indicate that the EGFR/HER2 and downstream AKT/PI3K and ERK/MAPK signalling pathways often remain inhibited by RTK inhibitors in resistant cells, alternative redundant signalling routes are instead engaged and converge on re-activation of common downstream effectors^5^,^6^,^7^. Proposed bypassing routes include the activation of kinases downstream of β1 integrin^8^,^9^, stimulation of the mechanistic target of rapamycin (mTOR) complex 1 (mTORC1)^10^,^11^,^12^,^13^ and enhanced autocrine mitogenic signalling^14^,^15^,^16^,^17^,^18^. While these observations suggest that reactivation of RTK or of alternative signalling routes may restore the proliferative potential of lapatinib-treated breast cancer cells, the downstream effects responsible for conferring resistance to lapatinib remain unknown.

The metabolic status of cancer cells impacts on their response to drugs. Indeed, targeting glycolysis sensitizes HER2-positive cells to HER2 inhibition by Herceptin treatment^19^ and the redox status can predict the response to HER2-inhibiting drugs in breast cancer cells^20^. Furthermore, lapatinib-resistant breast cancer cells display upregulation of genes controlling the glucose deprivation response network, suggesting a potential influence of the metabolic state of the cell in the response to lapatinib^21^. However, the specific signalling pathways and transcriptional regulators responsible for the metabolic adaptations contributing to lapatinib resistance in breast cancer cells are undefined.

Oestrogen-related receptor α (ERRα, NR3B1), an orphan member of the superfamily of nuclear receptors^22^, is a master regulator of cellular energy metabolism in both normal and cancer cells^23^,^24^,^25^. ERRα expression positively correlates with HER2 status and with poor prognosis in breast tumours^26^,^27^, and we recently showed that it contributes to ERBB2-dependent mammary tumorigenesis in mice^28^. Mechanistically, EGF has been shown to induce the recruitment of ERRα to the promoter of the *TFF1* gene^29^, while signals from HER2 impact on ERRα transcriptional activity^30^,^31^. These observations suggest that ERRα could participate as a downstream effector of mitogenic signals to mediate the metabolic adaptation of HER2-positive breast cancer cells and subsequently in their response to lapatinib.

Here we explore the hypothesis that ERRα acts as an effector of mitogenic signalling responsible for metabolic adaptations of breast cancer cells and further provide evidence of its implication in the therapeutic response and resistance to the RTK inhibitor lapatinib.

## Results

### Growth factor-dependent activity of ERRα in breast cancer cells

**Figure 1:**
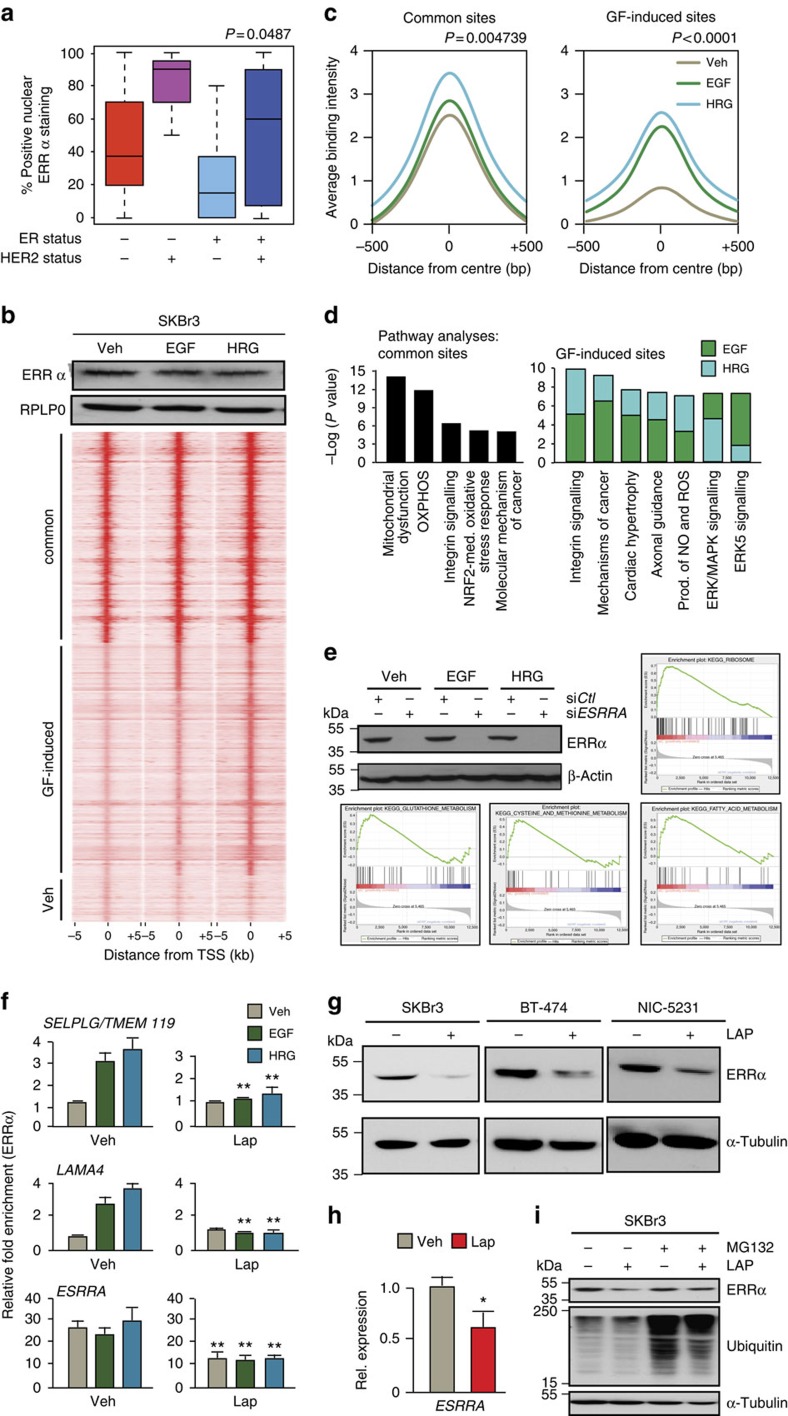
RTK-dependent ERRα activity in breast cancer cells. (**a**) Box plot depicting the % of nuclei positive ERRα by immunohistochemical staining in human breast tumour samples classified in various subtypes. Significance determined by one-way analysis of variance (ANOVA; *P*=0.0487). (**b**) Heatmap showing the intensities of ERRα binding of peaks determined by ChIP-seq in SKBr3 cells on 90 min stimulation with EGF (100 μM) or HRG (100 μM) or vehicle (veh), and clustered according to overlapping or growth factor-specific peaks by Homer. Below: western blot of ERRα and RPLP0 as loading control. (**c**) Average binding intensity of ERRα recruitment in the region ±500 bp around the centre of the peaks bound in all conditions (common sites) or induced on growth factor treatment (GF-induced). Statistical significance calculated by one-way ANOVA. (**d**) Enrichment of canonical pathway analysis classified according to –log(*P* value) generated by IPA for target genes displaying ERRα recruitment at ±20 kb from their transcriptional start site, in serum-starved conditions or on growth factor treatment. (**e**) Gene set enrichment of KEGG pathways enriched on depletion of ERRα in SKBr3 cells and specifically observed on treatment of cells with growth factors (false discovery rate<25%). siRNA-mediated depletion of ERRα is monitored by western blot analysis in all the conditions studied (upper left panel). (**f**) Standard ChIP analysis of genomic enrichment of ERRα recruitment to growth factor-reprogrammed sites in SKBr3 cells on treatment with vehicle or lapatinib. (**g**) Western blot depicting the expression of ERRα in SKBr3, BT-474 and NIC-5231 breast cancer cells on treatment with lapatinib. (**h**) Relative mRNA expression of *ESRRA*, a known target gene of ERRα in SKBr3 cells on lapatinib treatment. (**i**) Expression of ERRα and ubiquitin in SKBr3 cells treated with lapatinib or veh in the presence or not of the proteasome inhibitor MG-132. All statistical significance is calculated for results in lapatinib-treated cells relative to vehicle (veh) using three independent replicates. Error bars represent s.e.m., statistical significance is calculated using two-tailed unpaired *t*-test; *P<0.05; **P<0.01.

We previously showed that ablation of ERRα significantly delays ERBB2-induced tumour development in mice and lowers the levels of the ERBB2 amplicon transcripts^28^. To further investigate the interplay between RTK signalling and ERRα activity in breast cancer, we quantified the level of ERRα-positive nuclear staining in breast tumour samples from various subtypes using a specific antibody for ERRα (Supplementary Fig. 1a) and observed that the HER2-positive/oestrogen receptor-negative tumours express the highest level of ERRα-positive nuclei with a median expression of 90% (Fig. 1a, one-way analysis of variance *P* value=0.0487).

We then considered whether RTK signalling impacts on the genetic programmes regulated by ERRα in breast cancer cells. Serum-starved ERBB2-positive SKBr3 breast cancer cells were exposed to EGF or heregulin (HRG) to activate HER2 through EGFR/HER2 and HER2/HER3 signalling routes, respectively. The ERRα cistromes were generated using chromatin-immunoprecipitation (ChIP) followed by massive parallel sequencing (ChIP-seq). As shown in Fig. 1b,c, growth factor treatment induces a significant potentiation and reprogramming of ERRα binding without affecting the level of ERRα expression. A large number of genomic regions are significantly bound by ERRα only on growth factor treatment in SKBr3 cells (Fig. 1b,c and Supplementary Fig. 1b,d), including the region within the *TFF1* promoter originally described^29^ as an EGF-induced ERRα binding site in MCF-7 breast cancer cells (Supplementary Fig. 1b). Moreover, while ERRα binds to several common regions regardless of cell context, the addition of growth factors to the media significantly amplifies the signal intensity of ERRα recruitment to both these common sites and growth factor-specific sites (Fig. 1c and Supplementary Fig. 1c). Similar reprogramming of ERRα binding by ChIP-seq was observed in another HER2-amplified breast cancer cell line, BT-474, on growth factor treatment (Supplementary Fig. 1e). *De novo* binding site analyses identified the ERRα response element (ERRE: *Esrrb*, *Nr5a2* and *Erra*) as the most enriched motif in segments bound by ERRα both in serum-starved and growth factor-treated SKBr3 and BT-474 cells. However, treatment of both cell lines with EGF or HRG led to the specific co-enrichment of the binding sites for AP-1 (Jun/Fos) along with the ERRE in ERRα-bound segments (Supplementary Fig. 1f,g). These observations reveal a growth factor-dependent cooperation between ERRα and AP-1 in ERBB2-positive breast cancer cells.

We then assessed the biological significance of growth factor-dependent potentiation of ERRα binding in breast cancer cells through analysis of enriched functional pathways. Target genes harbouring ERRα-binding sites within 20 kilobases (kb) of their transcriptional start site, commonly bound in growth factor-treated cells and in untreated conditions, are involved in biological pathways related to previously reported functions of ERRα (ref. 32), including the control of mitochondrial metabolic functions and less-studied functions like stress response (Fig. 1d). The promoters bound by ERRα exclusively upon growth factor treatment are preferentially associated with genes regulating tumorigenic processes, including RTK and integrin signalling, proliferation and metastatic processes (Fig. 1d). To assess the functionality of this growth factor-modulated binding of ERRα, we further intersected our ChIP-seq data with gene expression data generated on depletion of ERRα in SKBr3 cells with and without growth factor treatment (Fig. 1e). While the depletion of ERRα in SKBr3 cells in all the conditions tested leads to altered regulation of target genes controlling mitochondrial metabolism (OXPHOS, tricarboxylic acid (TCA) cycle and glycolysis/gluconeogenesis) (Supplementary Fig. 1h and Supplementary Table 1), the depletion of ERRα in EGF- or HRG-treated cells also triggered the alteration of ERRα target genes controlling processes related to ribosome, fatty acid, glutathione, cysteine and methionine metabolism, as well as amino acid degradation (Fig. 1e and Supplementary Table 2). Moreover, the ERRα-binding intensity in genes regulating these metabolic functions or of genes in the glutathione/detoxification pathway (list adapted from ref. 33) is increased on growth factor treatment (Supplementary Fig. 1i). These results show that growth factor-induced RTK signalling in breast cancer cells potentiates the activity of ERRα to modulate the expression of target genes implicated in various aspects of cellular metabolism, including glutathione-mediated detoxification.

We further assessed whether pharmacological inhibition of EGF and HRG signalling could prevent this growth factor-dependent potentiation of ERRα recruitment to chromatin. SKBr3 cells were treated with the dual EGFR/HER2 inhibitor lapatinib to block growth factor signalling through EGFR/HER2/HER3. Standard ChIP experiments revealed that the binding of ERRα to growth factor-reprogrammed sites is abolished upon exposure to lapatinib (Fig. 1f, four upper panels). Unexpectedly, lapatinib treatment also triggered a significant decrease in ERRα recruitment to regions that are constitutively bound in absence of growth factors as depicted by the *ESRRA* promoter (Fig. 1f, lower panels). This suggests that the inhibitory effect of lapatinib on ERRα recruitment to chromatin is not limited to growth factor-reprogrammed sites but could affect all sites bound by ERRα. We therefore monitored the expression of ERRα in SKBr3 cells and observed that 24-h lapatinib treatment induces a sharp decrease in ERRα protein in SKBr3 and BT-474 cells, as well as in the mouse cell line NIC-5231 derived from ErbB2-driven mammary tumorigenesis^34^ (Fig. 1g). Quantitative PCR analyses revealed a similar decrease in *ESRRA* mRNA levels (Fig. 1h), likely a consequence of the decrease in ERRα protein levels since ERRα autoregulates its own expression^35^. Treatment of SKBr3 cells with the potent proteasome inhibitor MG-132, which blocks ubiquitin-conjugated protein degradation, prevented the lapatinib-dependent degradation of ERRα (Fig. 1i), indicating that the effect of lapatinib on ERRα protein levels occurs at least in part through proteosomal degradation. Taken together, these results reveal that growth factor-induced signalling potentiates ERRα transcriptional activity in regulating cellular metabolism and oxidative stress response and that inhibition of this signalling with RTK inhibitors leads to the degradation of ERRα.

### Re-expression of ERRα in lapatinib-resistant cells by mTOR

**Figure 2:**
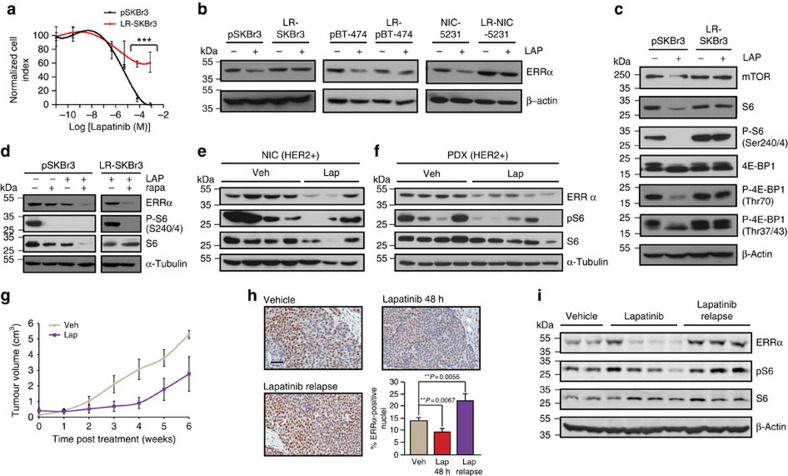
mTOR-dependent re-expression of ERRα in lapatinib-resistant cells. (**a**) Dose-dependent curves of normalized cell index for pSKBr3 and for lapatinib-resistant SKBr3 (LRSKBr3) cells on C29 treatment and exposed to varying concentration of lapatinib. Error bars are s.d.'s from three independent replicates, statistical significance is calculated in C29-treated cells relative to vehicle using unpaired Student's *t*-test (**P<0.01; ***P<0.001). (**b**) Expression of ERRα or veh treatment (left panel). Expression of ERRα in parental (pSKBr3) and in LRSKBr3 cells, in parental and in lapatinib-resistant BT-474 and in parental (NIC-5231) and in resistant cells on 24-h lapatinib or veh treatment. (**c**) Expression of mTOR, S6, P-S6, 4E-BP and P-4E-BP in pSKBr3 and LRSKBr3 cells on 24-h lapatinib or veh treatment. (**d**) Expression of ERRα, S6 and P-S6 in pSKBr3 (left) and LRSKBr3 (right) cells on 24-h lapatinib or veh treatment in presence or absence of rapamycin treatment. (**e**) Expression of ERRα in ERBB2-dependant mouse mammary NIC tumours following acute treatment with lapatinib for 48 h (left panel). (**f**) Expression of ERRα in HER2-positive PDX tumour following treatment with lapatinib for 48 h. (**g**) Growth curves representing the tumour volume of ERBB2-dependant mammary NIC tumors upon long-term lapatinib (*n*=6) or veh (*n*=5) treatment. Error bars are SEM. (**h**) Immunohistochemical (IHC) staining of ERRα expression in NIC tumours. Bottom right panel—quantification of IHC staining. Scale bar, 50 μM. Error bars are s.e.m., *P* values generated using unpaired Student's *t*-tests. (**i**) Expression and activity of ErbB2 and downstream effectors in ErbB2-dependant NIC mouse mammary tumours following acute treatment with lapatinib for 48 h and on lapatinib relapse.

On the basis of these findings, we next investigated whether ERRα could play a role in the resistance to lapatinib treatment in breast cancer cells. SKBr3, BT-474 and NIC-5231 cells were made resistant to lapatinib on long-term exposure to gradually increasing doses of lapatinib as previously described^36^. We confirmed that lapatinib-resistant SKBR3 (LRSKBr3) cells survive in higher doses of lapatinib than parental cells (pSKBr3) (Fig. 2a). In contrast to parental cell lines where lapatinib treatment decreases ERRα protein levels, the expression of ERRα is unaffected by lapatinib treatment in LRSKBr3 cells, as well as in the lapatinib-resistant BT-474 (LR-BT-474) and NIC-5231 (LR-NIC-5231) cells (Fig. 2b).

To gain insight into the mechanisms leading to ERRα re-expression in lapatinib-resistant cells, we monitored the status of RTK and downstream effectors involved in EGFR/HER2/HER3 signalling. We first confirmed that acquired lapatinib resistance in LRSKBr3 cells does not lead to reactivation of EGFR/HER2/HER3 or of downstream signalling pathways in LRSKBr3 cells as monitored by the phosphorylation status of the receptors and their downstream effectors (Supplementary Fig. 2a). We next assessed the status of the energy sensors AMPK and mTOR acting downstream of RTK signalling. While the levels of total AMPK are stable, phosphorylated AMPK, which is triggered in response to stressful stimuli, including oxidative stress, increases on lapatinib treatment both in parental and resistant cells, supporting the efficacy of the treatment in both cell lines (Supplementary Fig. 2b). However, while lapatinib treatment in pSKBr3 cells induces a decrease in total mTOR protein levels and in the levels of the activated forms of its downstream phosphorylated effectors P-S6 and P-4E-BP1 (Fig. 2c), mTOR signalling is insensitive to the inhibitory action of lapatinib in resistant cells (Fig. 2c). This suggests that reactivation of mTOR activity becomes decoupled from EGFR/HER2/HER3 and AMPK signalling in lapatinib-resistant cells.

We next tested the potential involvement of the mTOR pathway in the re-expression of ERRα in lapatinib-resistant cells. As shown in Fig. 2d, co-treatment of parental SKBr3 cells with the mTOR inhibitor rapamycin exacerbates the lapatinib-mediated decrease in ERRα levels. While high levels of ERRα and P-S6 are observed in resistant cells treated with lapatinib, co-treatment with rapamycin induces a significant decrease in the levels of these proteins in resistant cells (Fig. 2d), indicating that mTOR re-activation in lapatinib-resistant cells is linked to ERRα re-expression. We observed a similar effect with the mTORC1-specific inhibitor INK1341 that also induces a decrease in ERRα levels in lapatinib-resistant SKBr3 cells (Supplementary Fig. 2c). Similarly, SKBr3 cells resistant to treatment with the HER2-specific antibody trastuzumab also display increased levels of ERRα protein and activation of mTOR signalling through an increase in P-S6 levels when compared with parental cells. This suggests that reactivation of ERRα expression via mTOR signalling plays a key role in the development of HER2 targeted drug resistance in distinct therapeutic contexts (Supplementary Fig. 2d).

We next assessed whether the inhibitory effect of lapatinib on ERRα expression also occurs *in vivo* using both a mouse model of human HER2-driven breast cancer (MMTV-NIC)^37^ and HER2-positive patient-derived xenografts (PDXs) propagated in NOG mice. Following acute treatment of the mice with lapatinib or vehicle for 48 h before harvesting, we observed a considerable decrease in the expression of ERRα in the lapatinib-treated NIC and PDX tumours (Fig. 2e,f). As observed in the cell line models, this decrease in ERRα levels was concomitant with the inhibition of mTOR signalling on lapatinib treatment *in vivo* as assessed by the reduction in the phosphorylation of the mTOR effector P-S6 (Fig. 2e,f).

We further investigated whether the expression of ERRα was also reactivated *in vivo* in tumours that have acquired the capacity to grow in the presence of lapatinib. Mice bearing ERBB2-dependent MMTV-NIC mammary tumours were treated with lapatinib or vehicle using previously published protocols^38^,^39^ for a period of 6 weeks. Despite an initial response and tumour volume stabilization in the lapatinib-treated mice, the tumours eventually relapsed and acquired the ability to grow in the presence of the drug (Fig. 2g). As observed in cell culture models, monitoring the expression of ERRα by immunohistochemical staining and immunoblotting revealed a significant decrease of ERRα expression on acute lapatinib treatment compared with the control treatment arm, and an increase at end point in ERBB2-dependent tumours that have relapsed (Fig. 2h,i). Further, decreased ERRα expression on acute lapatinib treatment *in vivo* in the NIC tumours is concomitant with decreased mTOR signalling, while lapatinib-resistant tumours exhibit restoration of mTOR signalling along with the re-expression of high levels of ERRα (Fig. 2i). These results suggest that development of lapatinib resistance in ERBB2-driven mammary tumours *in vivo* recapitulates the mechanisms observed in the cell line models.

### ERRα dictates a metabolic signature in lapatinib-resistant cells

**Figure 3:**
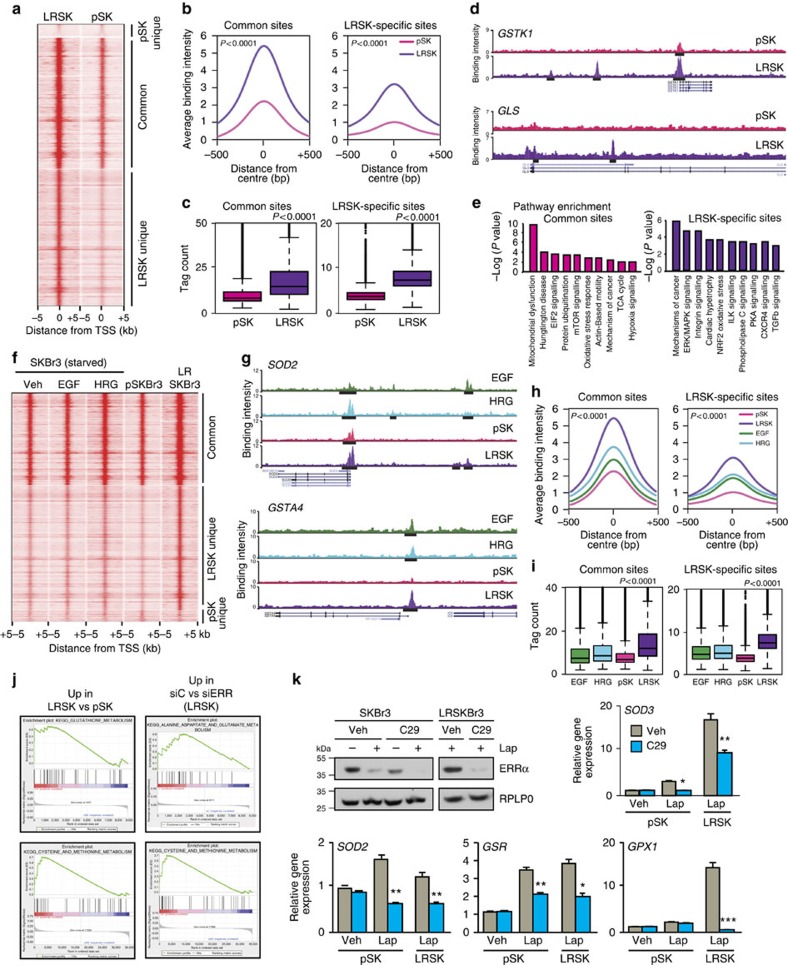
Reprogramming of ERRα activity in lapatinib-resistant cells. (**a**) Intensities of ERRα-binding events in LRSKBr3 and pSKBr3 cells. Segments are clustered according to peak detection. (**b**) Average binding intensity of ERRα recruitment in the region ±500 bp around the centre of the peaks in both pSKBr3 and LRSKBr3 cells (left panel) or in sites bound only in LRSKBr3 cells (right panel). Statistical significance calculated by standard unpaired *t*-test. (**c**) Distribution of tag counts across the peaks determined by ERRα ChIP-seq both in parental and resistant SKBr3. Statistical significance calculated by standard unpaired *t*-test. (**d**) Binding profiles for ERRα on lapatinib-resistant-reprogrammed sites in SKBr3 cells treated with EGF or HRG, and in pSKBr3 cells and LRSKBr3 cells maintained in lapatinib. (**e**) Enrichment of canonical pathway analysis generated by IPA for target genes displaying ERRα recruitment at ±20 kb from their transcriptional start site. (**f**) Intensities of ERRα-binding events in SKBr3 cells for each peak determined from ERRα ChIP-seq on veh, EGF or HRG, and in pSKBr3 cells and LRSKBr3 cells maintained in lapatinib and clustered according to peaks commonly bound by ERRα of in parental and resistant cells or uniquely bound in LRSKBr3 cells. (**g**) Binding profiles for ERRα in lapatinib-resistant-reprogrammed sites located in promoters and enhancers of genes regulating glutathione metabolism, in SKBr3 cells treated with EGF or HRG and in pSKBr3 cells and LRSKBr3 cells maintained in lapatinib. (**h**) Average binding intensity of ERRα recruitment in the region ±500 bp around the centre of the peaks bound by ERRα in both pSKBr3 and LRSKBr3 cells (left panel) or in sites bound only in LRSKBr3 cells (right panel). Statistical significance calculated by one-way analysis of variance (ANOVA). (**i**) Distribution of tag counts across the different peaks determined by ERRα ChIP-seq in parental and resistant SKBr3 and in SKBr3 cells treated with growth factors. Statistical significance calculated by one-way ANOVA. (**j**) Gene set enrichment of KEGG pathways associated to genes differentially regulated between parental and resistant SKBr3 cells (left panel; false discovery rate (FRD)<25%). GSEA enrichment on depletion of ERRα in LRSKBr3 cells (right panel; FDR<25%). siRNA-mediated depletion of ERRα (upper left panel). (**k**) Expression of ERRα target genes involved in oxidative stress response and glutathione synthesis. Error bars are s.d. on three independent replicates, statistical significance is calculated using unpaired Student's *t*-test (**P<0.01; *P<0.05). Top left panel: western blot showing the decrease in ERRα expression on C29 treatment both in parental and resistant SKBr3 cells.

Re-expression of ERRα in resistant cells prompted us to investigate the ERRα transcriptional programme in LRSKBr3 cells. Using a standard ChIP approach, we observed constitutive recruitment of ERRα to growth factor-induced binding sites in serum-starved lapatinib-resistant cells, even in absence of growth factor stimulation (veh bars in Supplementary Fig. 3a). Growth factor stimulation did not further enhance the recruitment of ERRα to these growth factor-induced sites in resistant cells. We therefore performed ERRα ChIP-seq to compare the ERRα-binding profile of untreated pSKBr3 cells to that of LRSKBr3 cells maintained in lapatinib. ERRα is recruited to more sites in the lapatinib-treated resistant cells than in the untreated parental cells (Fig. 3a and Supplementary Fig. 3b). The binding intensity of ERRα at both common and specific sites is significantly increased in LRSKBr3 cells compared with pSKBr3 cells (Fig. 3b–d). Interrogation of *de novo* binding sites revealed enrichment of the ERRE motif both in pSKBr3 and in LRSKBr3 cells (Supplementary Fig. 3c). As observed with the growth factor reprogramming of ERRα-binding profiles in parental SKBr3 cells (Fig. 1), co-occurrence of the AP-1 motif is observed at ERRα-binding sites that are specific to the resistant cells (Supplementary Fig. 3c).

Similar to results obtained in growth factor-treated breast cancer cells, Ingenuity Pathway Analyses of enriched biological processes reveal that the genes whose promoters are bound by ERRα both in pSKBr3 and LRSKBr3 cells are enriched for functions related to energy metabolism (Fig. 3e). The genes with ERRα promoter recruitment exclusively in LRSKBr3 cells regulate proliferation, invasion, metastasis, growth factor signalling and oxidative stress response. Comparison of the gene ontology also reveals an overlap between the functions of the genes whose promoters are bound by ERRα specifically in LRSKBr3 cells with those bound uniquely on growth factor treatment in parental cells (Supplementary Fig. 3d). This prompted us to compare the binding profile of ERRα in LRSKBr3 cells to the growth factor-dependent binding profile of ERRα in SKBr3 cells. We observe that sites bound by ERRα in LRSKBr3 cells but not in pSKBr3 cells are also typically bound by ERRα specifically upon growth factor treatment in SKBr3 cells (Fig. 3f,g and Supplementary Fig. 3e,f). We also observe that ERRα-binding intensity in lapatinib-resistant cells is closer to that observed upon growth factor treatment than in parental cells (Fig. 3h,i). This indicates that re-expression of ERRα in resistant cells contributes to re-establishment of the metabolic signature prevailing in growth factor-treated cells.

We next asked whether re-expression of ERRα in lapatinib-resistant breast cancer cells also contributes to their drug-resistant phenotype. We performed gene expression profiling of LRSKBr3 and compared the data with gene expression profiles of pSKBr3 cells. The acquisition of lapatinib resistance in SKBr3 cells is characterized by the upregulation of genes involved in the control of cell cycle, DNA repair, metastasis, RTK signalling, hypoxia, oxidative stress response (Supplementary Table 3) and glutathione, cysteine and glutamine metabolism (Fig. 3j, left panels). Depletion of ERRα using specific siRNAs in LRSKBr3 cells induces the downregulation of genes controlling cancer signalling along with various metabolic functions, including amino-acid metabolism, acetyl-coA biosynthesis, oxidative stress response, detoxification and regulation of glutamate, cysteine and methionine metabolism (Fig. 3j, right panels) (Supplementary Table 4). Intersecting these gene expression profiles with ERRα-bound promoters in LRSKBr3 cells resulted in a subset of direct ERRα target genes that are oppositely modulated on depletion of ERRα and acquisition of lapatinib resistance in SKBr3 cells (Supplementary Fig. 3g,h and Supplementary Tables 5, 6 and 7). Pathway analyses reveal that these ERRα direct target genes control key metabolic functions, including oxidative stress response, reactive oxygen species (ROS) detoxification, xenobiotic response, RTK signalling and cellular metabolism, including cysteine and glutamate metabolism (Supplementary Fig. 3i). This observation prompted us to assess the expression of ERRα target genes involved in glutathione-mediated ROS detoxification in parental and resistant cells. We used pharmacological inhibition of ERRα activity using the specific inhibitor Compound 29 (C29)^40^ that leads to decreased ERRα expression in both cell lines (Fig. 3k). The expression of the detoxification enzymes *SOD2*, *SOD3*, *GSR* and *GPX1* is increased on lapatinib treatment in parental cells and in lapatinib-resistant cells maintained in the drug compared with parental cells (Fig. 3k). Inhibition of ERRα activity by C29 treatment leads to a significant decrease in lapatinib-induced expression of these detoxification target genes, both in lapatinib-sensitive and -resistant SKBr3 cells (Fig. 3k).

### ERRα drives the glutamine flux in lapatinib-resistant cells

We investigated the metabolic states prevailing in the resistant SKBr3 cells. We first assayed the flux of nutrients through glycolysis or glutamine oxidation using ^13^C6-glucose or ^13^C5-glutamine tracer analysis, respectively, on pharmacological inhibition of ERRα activity using C29. As shown in Supplementary Fig. 4, lapatinib treatment induces a decrease in glucose flux in pSKBr3 cells as assayed through ^13^C6-glucose incorporation into pyruvate and lactate m+3 metabolite pool levels. While lapatinib treatment has no effect on glucose flux in LRSK cells, ^13^C6-glucose tracer analysis shows that glucose flux is decreased in resistant cells compared with control-untreated parental cells (Supplementary Fig. 4), indicative of an incomplete re-establishment of the glucose flux in resistant cells. Pharmacological inhibition of ERRα activity by C29 treatment did not affect glucose flux in either parental or resistant cells, indicating that the lapatinib-induced decrease in glucose flux in parental cells is independent of ERRα.

**Figure 4:**
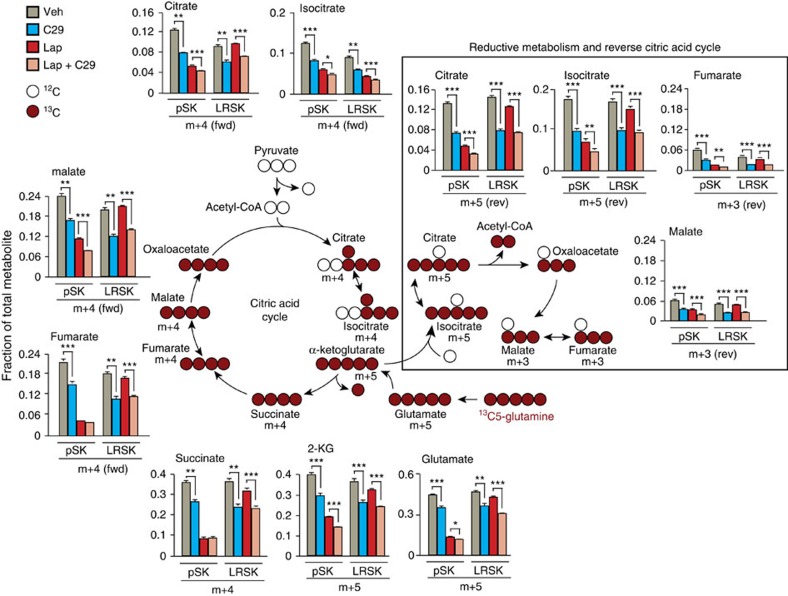
ERRα restores the glutamine flux in lapatinib-resistant cells. Schema representing the flux of ^13^C5-labelled glutamine through the citric acid cycle in forward and reverse directions. Isotope-labelled carbon is shown in red, and total change in metabolite mass is indicated. Bar charts show quantification of the fraction of intermediate metabolites produced from the flux of ^13^C5-labelled glutamine in pSKBr3 and LRSKBr3 cells on lapatinib treatment and pharmacological inhibition of ERRα with C29. Fraction of labelled intermediate metabolites issued from the TCA cycle is detected for both the forward (outside box) and reverse (inside box) cycle flux. Each graph represents three independent experiments performed in triplicate, error bars are s.e.m., statistical significance is calculated using two-tailed unpaired *t*-test; *P<0.05; **P<0.01; ***P<0.001. Western blot depicts the expression of ERRα in pSKBr3 and LRSKBr3 cells on 24-h lapatinib and/or C29 treatment.

Assessment of glutamine metabolism using ^13^C5-glutamine tracer analysis allows the study of both forward and reverse glutamine fluxes through the TCA cycle (Fig. 4)^41^. Lapatinib treatment significantly decreased glutamine flux in parental cells while it had only a moderate effect in lapatinib-resistant cells as determined for all the TCA cycle metabolites tested, both in the forward and reverse directions (Fig. 4). Importantly, we show that pharmacological inhibition of ERRα activity using C29 induces a significant decrease in glutamine flux, in both the parental and lapatinib-resistant SKBr3 cells, upon either control or lapatinib treatment. This result indicates that re-expression of ERRα in the lapatinib-resistant cells is essential for re-instatement of glutamine metabolism.

### Suppression of ERRα re-sensitizes resistant cells to lapatinib

**Figure 5:**
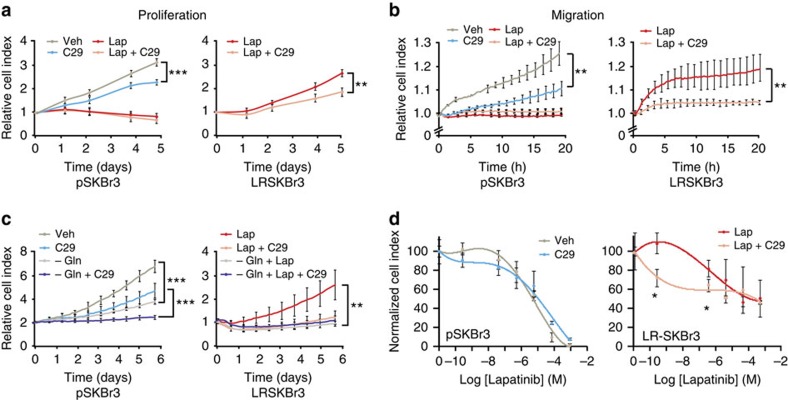
Suppression of ERRα and glutamine sensitize cells to lapatinib. (**a**) Normalized cell index curves representing proliferation in pSKBr3 (left panel) and LRSKBr3 (right panel) cells on treatment with lapatinib and in presence or absence of the ERRα inhibitor C29 treatment. (**b**) Migration curves in pSKBr3 (left panel) and LRSKBr3 (right panel) cells on lapatinib and C29 treatment. (**c**) Normalized cell index curves representing proliferation in pSKBr3 (left panel) and LRSKBr3 (right panel) cells on depletion of L-glutamine from the growth media in presence or absence of the ERRα inhibitor C29. (**d**) Normalized cell index curves representing the survival of parental (left panel) and lapatinib-resistant (right panel) SKBr3 cells on increasing doses of lapatinib treatment. All error bars are s.d. from three independent experiments, statistical significance is calculated with a two-tailed unpaired *t*-test; *P<0.05; **P<0.01; ***P<0.001.

Given the involvement of ERRα in the genomic reprogramming of ERRα binding to promoters of genes involved in tumorigenic processes in lapatinib-resistant cells, we tested whether inhibiting ERRα activity could affect the proliferative and migratory potential of pSKBr3 and LRSKBr3 cells. Pharmacological inactivation of ERRα using C29 leads to a decrease in proliferation and migration of sensitive cells (Fig. 5a,b, left panels). While LRSKBr3 cells kept proliferating and migrating on exposure to lapatinib, pharmacological inhibition of ERRα activity using C29 led to a significant decrease in proliferation and migration of the resistant cells (Fig. 5a,b, right panels).

Given the effect of ERRα inhibition on glutamine metabolism, we investigated the effect of glutamine deprivation from the media in both parental and resistant SKBr3 cells. Glutamine deprivation significantly decreased the proliferation of sensitive SKBr3 cells (Fig. 5c). Treatment with C29 further potentiated the effect of glutamine deprivation on proliferation in the sensitive cells. In lapatinib-resistant cells, glutamine deprivation lead to a drastic and significant decrease in proliferation, suggesting that inhibition of glutamine utilization can re-sensitize the cells to lapatinib treatment.

Since the re-expression of ERRα in the resistant cells contributes to the re-instatement of glutamine metabolism, we assessed whether inhibition of ERRα contributes to re-sensitization of the cells to lower doses of lapatinib. We analysed lapatinib sensitivity in SKBr3 cells upon treatment with C29. While inhibition of ERRα activity with C29 had no significant effect on the lapatinib sensitivity of parental SKBr3 cells, inhibition of ERRα in the resistant cells contributed to the re-sensitization of the LRSKBr3 cells to lower doses of lapatinib treatment (Fig. 5d).

### ERRα-driven metabolic adaptations restore detoxification capacity

**Figure 6:**
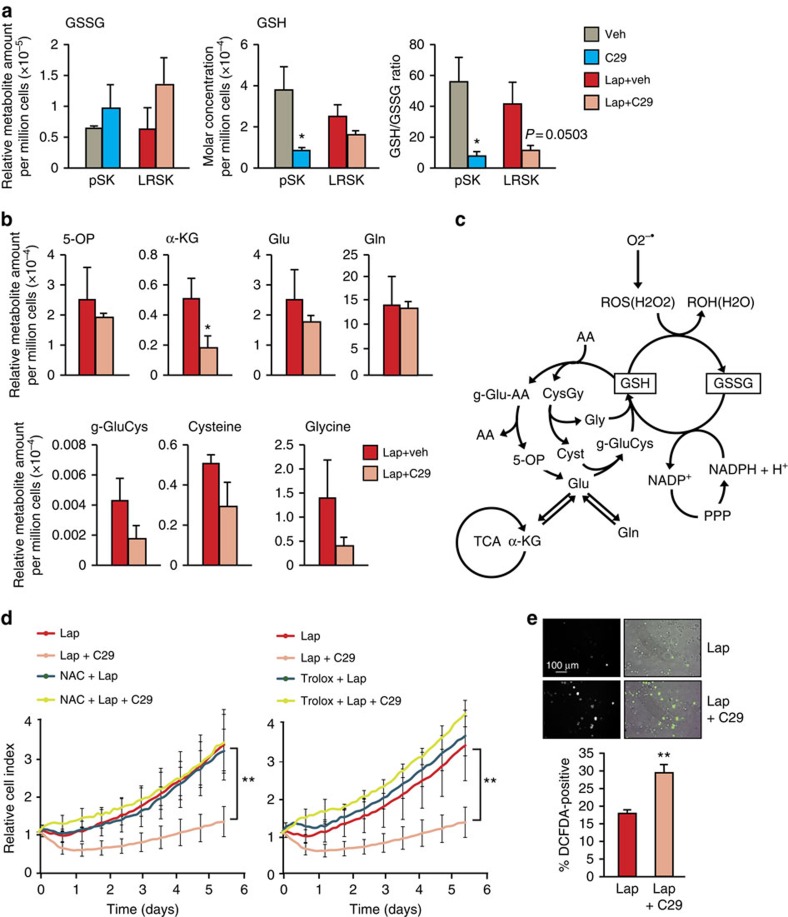
ERRα-driven metabolic adaptations restore detoxification capacity. (**a**) Quantification of the absolute and relative levels of GSH/GSSG in pSKBr3 or LRSKBr3 cells on lapatinib and C29 treatment. (**b**) Quantification of the absolute and relative levels of intermediate metabolites of the glutathione synthesis pathway in pSKBr3 or LRSKBr3 cells on lapatinib and C29 treatment. (**c**) Schematic diagram illustrating glutathione metabolism and ROS detoxification in relation to other metabolic pathways. Green, downregulated on C29 treatment in LRSKBr3 cells; red, upregulated; blue, unchanged. (**d**) Normalized cell index curves representing the survival of lapatinib-resistant SKBr3 cells on treatment with the antioxidant compounds *N*-acetylcysteine (NAC; 500 μM) or Trolox (500 μM) in presence or absence of the ERRα inhibitor C29. (**e**) Live imaging (top panel) and quantification (bottom panel) of lapatinib-resistant SKBr3 cells stained using DCFDA to detect ROS in the presence of lapatinib alone or in combination with the ERRα inhibitor C29. All error bars are s.d. from three independent experiments, statistical significance is calculated using two-tailed unpaired *t*-test; *P<0.05; **P<0.01.

Since the acquisition of lapatinib resistance in SKBr3 cells is characterized in part by ERRα-dependent re-establishment of glutamine metabolism and by an ERRα-target gene signature controlling oxidative stress response and ROS detoxification, we assessed whether re-expression of ERRα in lapatinib-resistant cells could also confer detoxification capacities enabling survival under lapatinib-induced oxidative stress conditions. We first demonstrated that a fraction of the pool of glutamine is used for generation of glutathione in the pSKBr3 (Supplementary Fig. 5). We next monitored the detoxification capacity of the cells by assessing the reduced versus oxidized glutathione (GSH/GSSG) ratio. We observed that inhibition of ERRα activity using C29 treatment leads to decreased GSH and increased GSSG levels, with an overall decrease in the GSH/GSSG ratio, indicative of reduced detoxification capacity and increased oxidative damage (Fig. 6a). Furthermore, we observed a reduction in the steady-state levels of several metabolites involved in the glutamine–glutathione biosynthesis pathway on C29-mediated inhibition of ERRα activity in lapatinib-resistant SKBr3 cells (Fig. 6b,c).

We further assessed whether the sustained expression of ERRα in the lapatinib-LRSKBr3 cells contributes to the detoxification potential of resistant cells using antioxidants treatment to counteract the effect of C29. Treatment of the cells with the antioxidants *N*-acetylcysteine and 6-hydroxy-2,5,7,8-tetramethylchroman-2-carboxylic acid (Trolox) rescued the survival of the lapatinib-resistant cells in the context of ERRα inhibition by C29 (Fig. 6d). We next assessed whether ERRα directly contributes to controlling the levels of ROS produced in the lapatinib-resistant cells. Staining and *in vivo* imaging quantification of LRSKBr3 cells using the ROS-detection probe DCFDA revealed that therapeutic inhibition of ERRα using C29 leads to a significant increase in the levels of DCFDA-positive cells, suggesting that the sustained expression of ERRα in the lapatinib-resistant cells contributes to ROS detoxification (Fig. 6e).

### Inhibition of ERRα impairs the growth of resistant tumours

**Figure 7:**
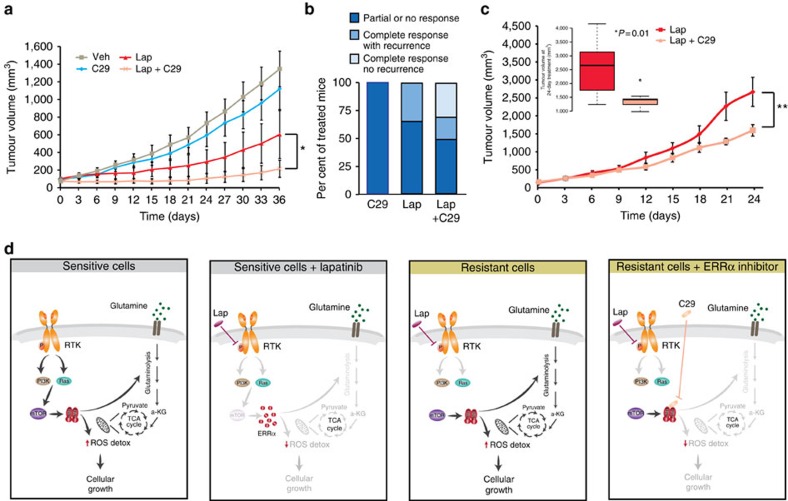
Inhibition of ERRα impairs the growth of lapatinib-resistant tumours. (**a**) Lapatinib-naive murine ErbB2-driven mammary tumours were transplanted into the mammary fat pads of MMTV-Cre mice. Growth curves indicate the response of the tumours to vehicle (*n*=8), C29 monotherapy (*n*=9), lapatinib monotherapy (*n*=12) and lapatinib/C29 combination therapy (*n*=10). *P<0.05, one-way analysis of variance (ANOVA) with Tukey's post-test. (**b**) Quantification of the therapeutic response and recurrence in response to sustained treatment with lapatinib and C29 individually and in combination. Partial or no response was defined as no effect of treatment on tumour growth or a reduced rate of growth with incomplete tumour regression. Complete response was defined as total regression of the tumour mass to a non-palpable state. Recurrence was defined as the regrowth of any palpable tumour mass within the follow-up period of 20 weeks. (**c**) A lapatinib-resistant murine ErbB2-driven mammary tumour was transplanted into the mammary fat pads of MMTV-Cre mice. Growth curves indicate the response of the tumours to lapatinib monotherapy (*n*=8) and lapatinib/C29 combination therapy. **P<0.01, one-way ANOVA with Tukey's post-test. Box plot shows the tumour size for both treatment groups at the end of the experiment. *P* value was generated by comparing the mean tumour size using an unpaired Student's *t*-test. (**d**) A working model of the role of the mTOR-ERRα axis in dictating the metabolic response of ERBB2-driven breast cancer cells to lapatinib and the potential for ERRα antagonists to counteract lapatinib resistance in those cells.

To determine the relevance of these observations to the growth of ErbB2-driven mammary tumorigenesis *in vivo*, we transplanted parental (lapatinib-sensitive) ErbB2-dependent NIC cells into the mammary fat pads of immunocompetent, coisogenic MMTV-Cre transgenic mice. These mice were then randomized to receive either vehicle, C29 alone, lapatinib alone or a combination of lapatinib and C29 treatment (Fig. 7a and Supplementary Fig. 6a). The growth of lapatinib-sensitive NIC tumours was monitored over a period of 36 days. As seen in the original transgenic mouse model (Fig. 2g), lapatinib treatment decreased tumour growth. While the inhibition of ERRα using C29 alone had little effect on tumour growth, addition of C29 to lapatinib treatment significantly decreased tumour growth compared with either treatment alone (Fig. 7a). Mammary tumours that underwent a complete response, as determined by complete regression of the tumour to a non-palpable state, inevitably recurred in all mice treated with lapatinib alone (Fig. 7b). However, the combination of C29 and lapatinib not only increased the proportion of complete responses but also prevented recurrence in 30% of treated mice over a follow-up period of 20 weeks. To determine whether ERRα inhibition could also affect the growth of tumours that had already acquired lapatinib resistance *in vivo*, we transplanted an MMTV-NIC ErbB2-driven mammary tumour that had relapsed following lapatinib treatment (Fig. 2g–i) into the mammary fat pads of immunocompetent, coisogenic MMTV-Cre transgenic mice. These mice were then randomized to receive either lapatinib alone or in combination with C29. The resistant NIC tumours grew robustly despite treatment with a high dose of lapatinib, reaching the maximum tumour volume within a 24-day period (Fig. 7c and Supplementary Fig. 6b). However, concomitant treatment with C29 markedly attenuated tumour growth, and the lapatinib/C29-treated tumours were significantly smaller at the end of the experiment (Fig. 7c, inset). Together, these results indicate an essential role for ERRα in establishing a favourable metabolic context that supports survival on lapatinib insult and favours proliferation of lapatinib-resistant cells, both *in vitro* and *in vivo*.

## Discussion

Mitogenic signalling is a prominent driver of breast tumorigenic processes, and resistance to TKIs is a common issue in the treatment of HER2-amplified breast tumours in the clinical setting. In this work, the global metabolic regulator ERRα is first identified as a transcriptional mediator translating downstream mitogenic signals into a metabolic signature that is impaired on treatment with the RTK inhibitor lapatinib. The present study further uncovers a molecular mechanism sustaining resistance to lapatinib that involves the constitutive re-expression of ERRα through mTOR re-activation in resistant cells. Reactivation of ERRα transcriptional programmes further elicits a suitable metabolic context enabling cell survival despite constant exposure to lapatinib-induced inhibition of HER family RTK signalling. Importantly, this study demonstrates that pharmacological inhibition of ERRα prevents this metabolic adaptation and restores lapatinib sensitivity in resistant cells in culture and mammary tumours *in vivo* (Fig. 7d).

The data presented herein reveal that growth factor signalling modulates the transcriptional activity of ERRα at a genome-wide level in breast cancer cells. Since poor-prognosis breast tumours display enhanced mitogenic signalling and increased activity of ERRα, our data imply that ERRα mediates a specific transcriptional programme in response to growth factor/RTK signalling in poor-prognosis breast tumours. In addition, ERRα directly regulates the expression of the *ERBB2* gene in breast cancer cells in the absence of the oestrogen receptor^28^. Here we show that ERRα in turn regulates tumorigenic processes on stimulation of EGFR/HER2/HER3 signalling, supporting the existence of an ERRα/RTK feed-forward loop further enhancing the mitogenic effect of HER2 in HER2-amplified breast tumours.

The mechanisms underlying the development of TKI resistance in breast cancer cells are diverse, yet they all involve the engagement of survival signals redundant to those transduced by the targeted kinases^6^,^42^. Our observation that the transcriptional profiles of ERRα in lapatinib-resistant cells mirror those observed in growth factor-treated sensitive cells reveals that the resistant cells provide a context favouring a mitogenic-like state affecting ERRα activity despite the constant blockade of EGFR/HER2/HER3 signalling by lapatinib. The observation that re-expression of ERRα in lapatinib-resistant cells depends on mTOR reactivation also denotes an important role of ERRα in breast cancer biology. Re-activation of mTOR signalling has been described as a mechanism potentially contributing to the development of resistance to various TKIs in cancer cells but the downstream mechanisms by which it does so have been largely unknown. The results presented here describe the downstream effects of constitutive mTOR reactivation and support a model by which the consequential re-expression of ERRα, occurring independently of EGFR/HER2/HER3 and downstream effectors signalling, can mediate at least a subset of the mitogenic effects and mTOR functions in resistant cells, despite the sustained inhibition of RTK signalling.

ERRα is a master regulator of energy and cellular metabolism. We demonstrate that lapatinib affects glucose and glutamine fluxes in breast cancer cells. Our observations indicate that re-expression of functional ERRα is required for optimal restoration of glutamine metabolism in resistant cells and that pharmacological inhibition of ERRα activity prevents this metabolic adaptation and desensitizes the cells to lapatinib treatment. Moreover, our data indicate that the constitutive re-expression of ERRα in resistant cells can restore the detoxification capacities and can prevent an increase in the levels of ROS, sustaining cell survival despite the oxidative stress conditions triggered on therapeutic insult (Fig. 7). Several of the genes regulating the oxidative stress response, which have previously been reported as upregulated in lapatinib-resistant cells^21^, are indeed direct targets of ERRα. In addition, the ERRα coactivator PGC-1α was shown to confer improved mitochondrial metabolism and detoxification capacities enabling survival under oxidative stress conditions in aggressive melanoma tumours^43^, and to determine a metabolic transcriptional programme promoting invasion and metastasis in breast cancer^44^. Our functional genomic and metabolomic data coupled with expression profiling suggest that these PGC-1α effects are likely mediated through ERRα in breast cancer cells since the re-expression of ERRα in resistant cells provides the optimal metabolic context required to survive the lapatinib-induced oxidative stress insult.

Importantly, our study suggests that pharmacological inhibition of ERRα activity represents a viable mechanism to counteract lapatinib resistance in breast cancer and to impact on metabolic adaptations occurring in resistant tumours. Our work also demonstrates the value of ERRα inhibition in the context of lapatinib treatment since co-treatment prevented tumour recurrence in 30% of treated mice. This study also supports the value of recent clinical trials assessing the therapeutic benefits of concomitant inhibition of mTOR and of RTK signalling in breast tumours^45^. Our work indicates that targeting further downstream effectors through inhibition of ERRα activity might represent an even more efficient way to impinge on metabolic adaptations triggered by mTOR reactivation. Taken together, our study demonstrates that modulation of ERRα promotes metabolic vulnerabilities that can be exploited therapeutically and supports inhibition of ERRα as an adjuvant therapy in advanced HER2-positive breast cancer.

## Methods

### Immunohistochemistry

MCF-7 cells were pelleted and re-suspended in Matrigel that was either formalin-fixed and processed for paraffin embedding/sectioning or flash-frozen in optimal cutting temperature (OCT) compound. OCT-embedded MCF-7 pellets and frozen breast tumours (ethical clearance) were fixed in a formalin/phosphate buffer (10%, 30 min). All sections were processed for antigen retrieval in heated TET buffer (10 mM Tris, 1 mM EDTA and 0.05% Tween 20, pH 9.0) for 15 min, and ERRα (using the anti-ERRα rabbit monoclonal antibody (Abcam 2131-1; 1:100 dilution)) expression was assessed using the biotin–streptavidin–peroxidase method. Expert pathologist scored epithelial zones according to their nuclear staining (%) and data were analysed using R-Bioconductor package using the boxplot function (R Core Team (2014; http://www.R-project.org/). For mouse tissues, 5-μm sections of formalin-fixed, paraffin-embedded murine mammary tumours were immunohistochemically stained with ERRα antibody (Epitomics; 1:100 dilution).

### Cell culture and antibodies

For all experiments, MCF-7, SKBr3, BT-474, pSKBr3 and LRSKBr3 cells were cultured as described previously^46^ and maintained in DMEM supplemented with 10% fetal bovine serum (FBS) and routinely tested for mycoplasma. Parental cells were obtained from American Type Culture Collection. Lapatinib-resistant cells were derived as previously described^47^ and continuously cultured in the presence of 2 μM lapatinib (LC Laboratories, L-4804). NIC-5231 and LRNIC-5231 cells were maintained in DMEM supplemented with 5% FBS, 5 ng ml^−1^ EGF, 1 μg ml^−1^ hydrocortisone, 5 μg ml^−1^ insulin (all from Wisent) and 35 μg ml^−1^ Bovine Pituitary Extract (Hammond Cell Tech). For acute lapatinib treatment, resistant cells were transferred to DMEM without lapatinib supplementation 24 h before treatment, and pSKBr3 and LRSKBr3 cells were treated with 2 μM lapatinib or dimethylsulphoxide (DMSO; veh) 24 h before harvesting. C29 treatment was carried on at a concentration of 5 μM for 24 h. For rapamycin (Calbiochem 553211) and INK1341 treatments, cells were treated for 48 h before treatment with rapamycin or DMSO (veh). For growth factor treatment, the cells were transferred to serum-deprived DMEM 24 h before treatment with 100 μM EGF (Sigma-Aldrich) or HRG (R&D Systems) or PBS (veh). For proteasome inhibitor experiments, cells were treated with 20 μM MG-132 24 h before harvesting. Pools of siRNAs against ERRα (si*ESRRA*), and control (siC) (ON-Target-Plus siRNA pool) were obtained from Dharmacon. Transfections were performed with Hiperfect (Qiagen). ERRα ChIP assays were performed using an anti-ERRα rabbit monoclonal antibody (Abcam 2131-1) and control antibody anti-rabbit IgG antibody (SantaCruz sc-2027). Western blots on cell extracts were performed using the following antibodies: ERRα (Abcam 2131-1; 1:500 dilution); mTOR (Cell Signaling 2972; 1:500 dilution); S6 (Cell Signaling 2217; 1:1,000 dilution); p-S6 (Ser 240/244, Cell Signaling 2215; 1:1,000 dilution); p-4E-BP1 (Thr 37/46, Cell Signaling, 9459; 1:1,000 dilution); p-4E-BP1 (Thr70, Cell Signaling 9455; 1:1,000 dilution); 4E-BP (Cell Signaling, 9955; 1:1,000 dilution); AMPK (Cell Signaling 2532; 1:500 dilution); p-AMPK (Thr172, Cell Signaling, 2531; 1:200 dilution); α-tubulin (Cell Signaling 4967; 1:2,000 dilution); β-actin (Santa-Cruz sc47778; 1:2,500 dilution); RPLP0 (Abcam ab88872; 1:1,000 dilution); EGFR (Cell Signaling 2232; 1:1,000 dilution); p-EGFR (Tyr1173, Cell Signaling 4407; 1:1,000 dilution); HER2 (Santa Cruz sc-284; 1:500 dilution); p-HER2 (Tyr1221/1223 Cell Signaling 2249; 1:500 dilution); HER3 (Santa Cruz sc-4754; 1:1,000 dilution); p-HER3 (Tyr1289, Santa Cruz sc4791; 1:1,000 dilution); p42/44ERK (Cell Signaling 9102; 1:1,000 dilution); p-p42/44ERK (Tyr202/204 Cell Signaling 9106; 1:750 dilution); AKT (Cell Signaling 9272; 1:1,000 dilution); and p-AKT (Ser473 Cell Signaling 9271; 1:1,000 dilution).

### *In vivo* studies

MMTV-NIC and MMTV-Cre transgenic mice (FVB/N background) have been described previously^37^,^48^. Lapatinib (150 mg kg^−1^) and C29 (10 mg kg^−1^) were administered daily by oral gavage as suspensions in 0.5% hydroxypropylmethylcellulose/0.1% Tween 80 (Sigma). Mice were randomly assigned to treatment groups, and tumour size was assessed in a blinded fashion using calliper measurements. Lapatinib administration in MMTV-NIC mice: female MMTV-NIC mice on a pure FVB/N genetic background were monitored for spontaneous mammary tumour formation. These mice developed mammary tumours with an average latency of 124.6±15.3 days, in line with previously published observations^37^. Once tumours were measurable, mice were randomly assigned to receive lapatinib (150 mg kg^−1^) or vehicle (0.5% hydroxypropylmethylcellulose, 0.1% Tween 80 – Sigma) daily by oral gavage. Group sizes were *n*=5 for vehicle and *n*=6 for lapatinib. Tumours were measured weekly using calipers, and tumour volume was calculated using the formula: 4.18879 × (*L*/2) × (*W*/2)^2^. Drug administration and tumour measurement were performed by distinct individuals and those measuring the tumours and analysing the data were blinded to the treatment received by each mouse. Mice were killed when their total tumour burden reached 5 cm^3^, as per guidelines approved by the McGill University Animal Care Committee. Once all vehicle-treated mice had reached the tumour burden end point (6 weeks following the initiation of the experiment), the experiment was terminated. Lapatinib/C29 administration in an immune-competent allograft model: a mammary tumour in an MMTV-NIC mouse that had relapsed following 6 weeks of treatment with lapatinib was surgically excised and transplanted into the mammary fat pads of female MMTV-Cre mice on the same genetic background (FVB/N). This mouse strain was chosen for its immune toleration to Cre recombinase, which we have found to be immunogenic in non-transgenic FVB/N mice receiving MMTV-NIC tumour transplants. Once tumours had reached a size of 5 × 5 mm (65 mm^3^), the mice were randomly assigned for treatment with lapatinib (150 mg kg^−1^) or lapatinib (150 mg kg^−1^) plus C29 (10 mg kg^−1^) in 0.5% hydroxypropylmethylcellulose/0.1% Tween 80 by daily oral gavage as described above. Group sizes were *n*=8 for lapatinib monotherapy and *n*=9 for lapatinib+C29 combination therapy. Tumour measurements and volume calculations were performed in a blinded fashion as described above. The experiment was terminated after 24 days of treatment when the majority of mice receiving lapatinib monotherapy had reached the maximum tumour size for a single mass (1.5 mm^3^). Average tumour volumes for the two experimental groups at the end of the experiment were compared using an unpaired Student's *t*-test. All animal studies were approved by the McGill University Downtown Campus Animal Facility Care Committee (FACC), which is a branch of the McGill University Animal Care Committee (UACC).

### Patient-derived xenografts

All human participants provided informed consent for this study, and tissue was collected at the McGill University Health Centre in accordance with the protocols approved by the research ethics board (SUR-99-780). All animal studies were approved by the UACC (2014-7514). PDXs were developed from 1–8-mm^3^ fragments of freshly resected breast tumour, submerged in PBS:Matrigel (1:1) (Corning), and transplanted into the fourth mammary fat pad of 5–7 week SCID-beige mice (Charles River) housed under pathogen-free conditions. Tumours were measured twice per week using callipers and collected when the largest tumour dimension reached 10 mm. PDXs were passaged using fragments from collected tumours as described above.

### ChIP assays and ChIP-sequencing

For ChIP-sequencing analyses, chromatin was prepared from SKBr3 or BT-474 cells cultured for 24 h in serum-depleted media and further treated with 100 μM EGF or HRG for 90 min prior harvesting. The ChIP primers are listed in Supplementary Table 8. ChIP-seq analyses were performed as previously described^49^. The sequences generated from ChIP-seq were treated as previously described^49^. Briefly, sequences were aligned to the human genome database (Hg19) using BWA v0.5.9 (ref. 50). Peaks were called using MACS v1.4.1 software (mfold=10, 30; bandwidth=300; *P* value cutoff=1E-5)^51^. Peak annotation, tag directory, bed file generation, merging and *de novo* motif discovery were performed with the Homer package v3.1 (http://biowhat.ucsd.edu/homer/ngs/index.html). Tag heatmaps were obtained with Java TreeView (http://jtreeview.sourceforge.net/). Standard ChIP was performed as described previously^46^. Quantification of ChIP enrichment by real-time quantitative PCR was carried out using the LightCycler480 instrument (Roche). ChIPs are normalized against background enrichment on anti-IgG antibody ChIP control and enrichment on a negative control unbound region. Representative graph of three independent experiments performed in triplicates are shown. Statistical significance of standard ChIP is obtained with unpaired Student's *t*-tests. For ChIP-sequencing, a library of enriched DNA segment following the ChIP experiment was prepared according to the ChIP-sequencing library protocol by Illumina and as previously described. Following quality control with an Agilent Bioanalyser 2100 the libraries were sequenced using an Illumina HiSeq 2000 (McGill University and Genome Quebec Innovation Centre).

### Expression profiling and gene set enrichment

SKBr3 cells were maintained as described and transfected with siRNAs 60 h before collecting. Cells were treated with 100 μM EGF, HRG or veh for 3 h or with 2 μM lapatinib for 24 h before RNA extraction with the RNeasy Mini Kit (Qiagen) and reverse transcribed using Superscript II (Invitrogen) and analysed by quantitative RT–PCR with SYBR-green (Roche). For expression profiling, RNA was hybridized to Illumina HT12-v4 Expression BeadChIP array according to the manufacturer's protocol. Expression data were normalized using the lumi normalization function in FlexArray (http://genomequebec.mcgill.ca/FlexArray). Differential gene expression was computed using the SAM function in FlexArray, and statistical test for fold changes were performed using Student's *t*-tests. Normalized data and differentially expressed genes were used as input for determining gene set enrichment analysis (GSEA) using the GSEA software^52^,^53^ and Ingenuity Pathways Analysis software using canonical pathways (Ingenuity(R) Systems, (Ingenuity(R) Systems, http://www.ingenuity.com/ (IPA)).

### Ingenuity pathway analyses for ChIP-seq

ERRα ChIP-seq target genes found within ±10 kb of binding site were analysed for significant biological pathways using Ingenuity Pathways Analysis software. Fisher's exact test was used to calculate *P* values representing the probability of association between genes in the data set and the canonical pathways.

### Cell proliferation and migration assays

For cell proliferation assays, cells were maintained as described above, seeded in 24-well plates and treated with 5 μM C29 or veh for 24, 48, 72 or 96 h. Cell viability was assessed using crystal violet as previously described^28^. For migration assays, cells were transferred to CIM-16 migration plates (Roche). Cells were allowed to migrate towards FBS and were monitored in real time using Xcelligence RTCA DP instrument (Roche). Statistical significance was calculated using Student's *t*-test and analysis of variance. For glutamine deprivation, cells were seeded in 96-well plates in either complete media or in glutamine-depleted media (+10% dialysed serum). Next day, cells were treated with 5 μM C29 or vehicle and transferred to IncuCyte ZOOM Live cell analysis system (Essen BioScience) and proliferation was monitored using IncuCyte ZOOM phase-contrast quantification software (2014A). For antioxidant treatments, resistant cells were plated in 96-well plates in presence of lapatinib. The next day, cells were treated with either 500 μM *N*-acetylcysteine or 500 μM 6-hydroxy-2,5,7,8-tetramethylchroman-2-carboxylic acid (Trolox), in presence or absence of 5 μM C29. The cells were transferred to the IncuCyte ZOOM Live cell analysis system (Essen BioScience) and quantified as described above.

### Live cell imaging and ROS quantification

Lapatinib-resistant SkBr3 cells were seeded in 24-well plates at a density of 2 × 10^4^ per well and treated with lapatinib alone (2 μM) or lapatinib+C29 (5 μM) for 48 h. Cells were then stained with 5 μM H2DCFDA (Thermo Fisher, D399) for 30 min under normal growth conditions and imaged in phenol-red free DMEM at 37 °C and 5% CO_2_ using a Zeiss Axiovert 200M equipped with a Zeiss LD A-Plan × 20 objective. Total and DCFDA-positive cells in a minimum of 10 independent fields (at least 1,000 total cells per condition) were quantified using ImageJ. The experiment was performed three times and statistical significance was determined using Student's *t*-test.

### GC/MS, LC/MS and mass isotopomer distribution analysis

Gas chromatography/mass spectrometry (GC/MS), liquid chromatography/MS (LC/MS) and Mass Isoprotomer Distribution analysis are described in Supplementary Methods. Briefly for GC/MS, pSKBr3 and LRSKBr3 cells were seeded in triplicate in 35-mm plates, grown to 50% confluence and treated with control (DMSO 0.1%) and/or Lapatinib and/or C29 for 24 h before changing media to 1 ml DMEM high glucose supplemented with 10% dialysed FBS (Wisent) and either 10 mM U-^13^C_6_-glucose (uniformly labelled, Cambridge Isotope Laboratories, CLM-1396, 99%) or 2 mM U-^13^C_5_-glutamine (Cambridge Isotope Laboratories, CLM-1822, 97-99%). After 30 min pulses, cells were collected and metabolites were derivatized using *N*-*tert*-Butyldimethylsilyl-*N*-methyltrifluoroacetamide (MTBSTA, 394882, Sigma) and subjected to GC/MS. For LC/MS analysis of glutamine flux into glutathione, parental and lapatinib-resistant SkBr3 cells were grown in DMEM containing 2 mM ^13^C_5_-labelled glutamine (CML-1822; Cambridge Isotope Laboratories, Inc.) or unlabelled glutamine for 6 h. Metabolites were then extracted for LC/MS and analysed as described in Supplementary Methods (Supplementary Tables 9 and 10). Matrix corrections for tracer analysis were carried out as described^54^.

### Data availability

All microarray and sequencing raw and processed data have been deposited in the GEO repository and are available under the accession numbers GSE81546 and GSE81651, respectively.

## Additional information

**How to cite this article:** Deblois, G. *et al*. ERRα mediates metabolic adaptations driving lapatinib resistance in breast cancer. *Nat. Commun.* 7:12156 doi: 10.1038/ncomms12156 (2016).

## Supplementary Material

Supplementary Information Supplementary Figures 1-6, Supplementary Tables 1-10, Supplementary Methods and Supplementary References.
