# Supplementary material for: ERRα mediates metabolic adaptations driving lapatinib resistance in breast cancer
Source: Nat Commun. 2016 Jul 12;7:12156. doi: 10.1038/ncomms12156 (PMC4945959; doi:10.1038/ncomms12156)
Supplement: Supplementary Information — Supplementary Figures 1-6, Supplementary Tables 1-10, Supplementary Methods and Supplementary References. [file ncomms12156-s1.pdf]

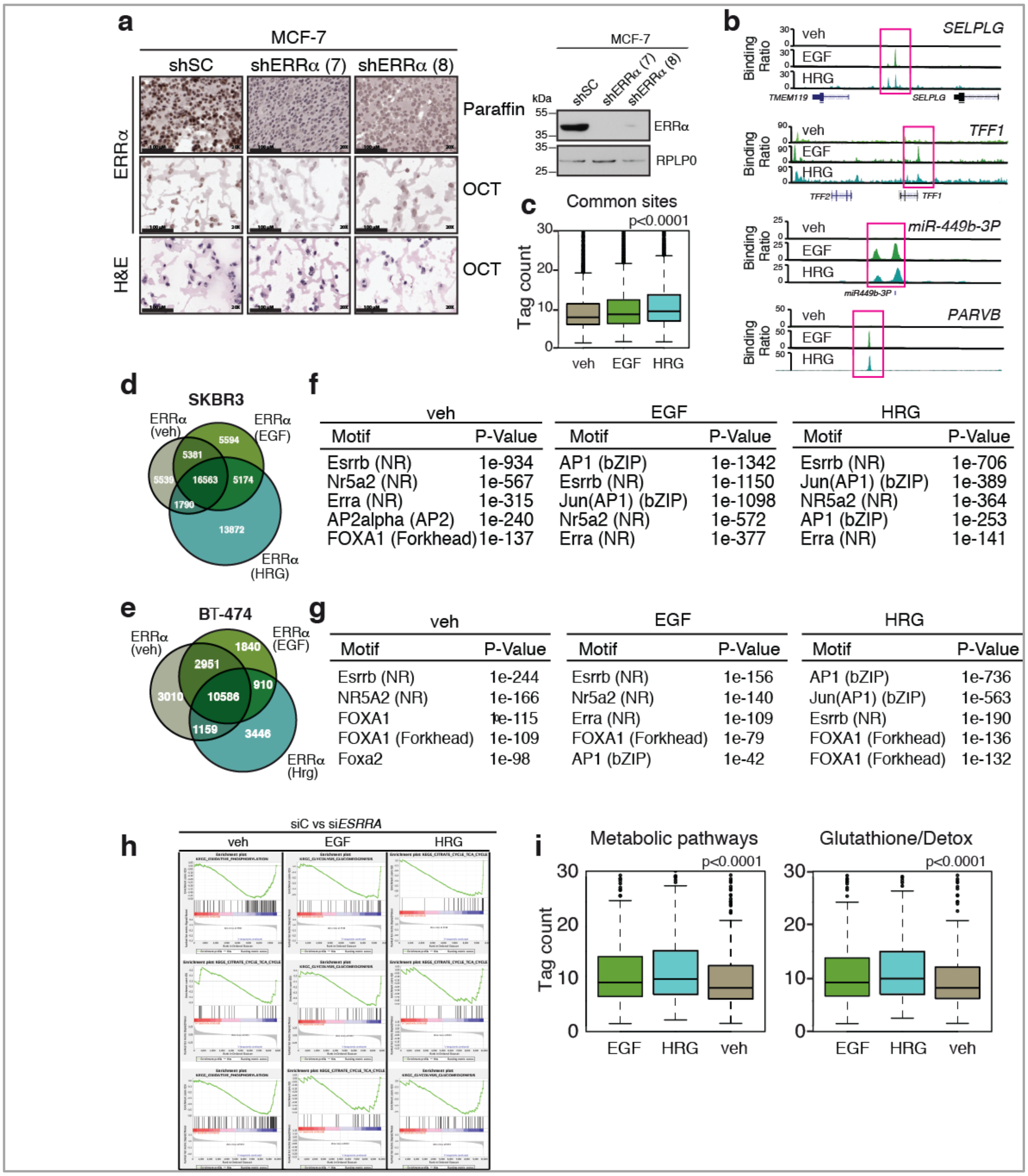
a
MCF-7
shSC
shERRα (7)
shERRα (8)
ERRα
H&E
Paraffin
OCT
OCT
MCF-7
kDa
shSC
shERRα (7)
shERRα (8)
55
35
35
25
ERRα
RPLP0
b
SELPLG
TFF1
miR-449b-3P
PARVB
Binding Ratio
veh
EGF
HRG
TMEM119
SELPLG
TFF2
TFF1
miR449b-3P
c
Common sites
p<0.0001
Tag count
veh
EGF
HRG
d
SKBR3
ERRα (veh)
ERRα (EGF)
ERRα (HRG)
5594
5381
5539
16563
5174
1790
13872
e
BT-474
ERRα (veh)
ERRα (EGF)
ERRα (Hrg)
1840
2951
910
3010
10586
1159
3446
f
veh
Motif P-Value
Esrrb (NR) 1e-934
Nr5a2 (NR) 1e-567
Erra (NR) 1e-315
AP2alpha (AP2) 1e-240
FOXA1 (Forkhead) 1e-137
EGF
Motif P-Value
AP1 (bZIP) 1e-1342
Esrrb (NR) 1e-1150
Jun(AP1) (bZIP) 1e-1098
Nr5a2 (NR) 1e-572
Erra (NR) 1e-377
HRG
Motif P-Value
Esrrb (NR) 1e-706
Jun(AP1) (bZIP) 1e-389
NR5a2 (NR) 1e-364
AP1 (bZIP) 1e-253
Erra (NR) 1e-141
g
veh
Motif P-Value
Esrrb (NR) 1e-244
NR5A2 (NR) 1e-166
FOXA1 1e-115
FOXA1 (Forkhead) 1e-109
Foxa2 1e-98
EGF
Motif P-Value
Esrrb (NR) 1e-156
Nr5a2 (NR) 1e-140
Erra (NR) 1e-109
FOXA1 (Forkhead) 1e-79
AP1 (bZIP) 1e-42
HRG
Motif P-Value
AP1 (bZIP) 1e-736
Jun(AP1) (bZIP) 1e-563
Esrrb (NR) 1e-190
FOXA1 (Forkhead) 1e-136
FOXA1 (Forkhead) 1e-132
h
siC vs siESRRA
veh
EGF
HRG
i
Metabolic pathways
Glutathione/Detox
p<0.0001
p<0.0001
Tag count
EGF
HRG
veh

Supplementary Figure 1 | Reprogramming of ERRα binding profile by growth factors. (**a**) Immunohistochemical analyses of ERRα of paraffin or OCT embedded MCF-7 clones stably transfected with shSC or two different shERRα. Western blot depicting the levels of ERRα upon shERRα-induced ERRα depletion is shown on the right panel. (**b**) Examples of binding profiles for ERRα on growth factor-reprogrammed sites in SKBr3 cells upon 90 min of EGF or HRG treatment. (**c**) Box plot representing the distribution of tag counts across the different peaks determined by ERRα ChIP-seq for each treatment conditions in SKBr3. Statistical significance calculated by one-way analysis of variance (ANOVA). (**d**) Venn diagram representing number of ERRα-bound segments obtained by ChIP-seq in SKBr3 cells. (**e**) *de novo* motif computational discovery in ERRα-bound sequences in SKBr3 cells in common or in EGF- and HRG-specifically bound segments. Enrichment of AP1 binding site specifically upon EGF or HRG treatment is highlighted in yellow. (**f**) Venn diagram representing number of ERRα-bound segments obtained by ChIP-Sequencing analyses in BT-474 cells upon 90 min stimulation with EGF or HRG. (**g**) *De novo* motif computational discovery in ERRα-bound sequences in BT-474 cells in total veh-treated or in EGF- and HRG-specifically bound segments. (**h**) Examples of top gene set enrichments from KEGG pathways by GSEA upon siRNA-mediated depletion of ERRα in all 3 treatment conditions (FDR<25%). (**i**) Box plot representing the distribution of tag counts across the different peaks for each treatment conditions in SKBr3 determined by ERRα ChIP-seq and falling within +/- 20 kb of the TSS of genes controlling cellular and energy metabolism (left panel, One-way Anova: $p<0.0001$) or glutathione and detoxification functions (right panel, one-way anova: $p<0.0001$). Gene lists filtered according to functions reported in ref. [1].

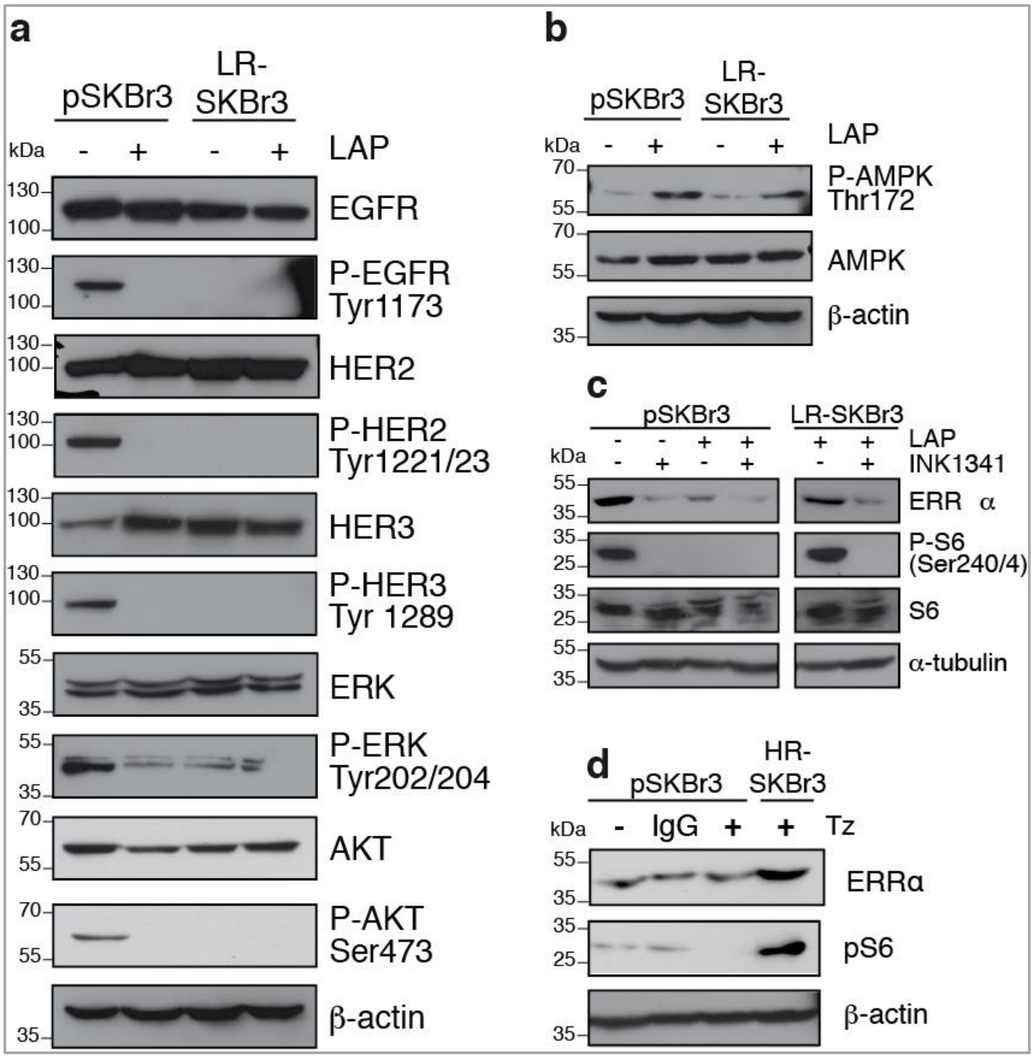

Supplementary Figure 2 | Growth factor signalling is affected in lapatinib-resistant cells. (**a**) Western blot showing the expression of basal and phosphorylated forms of EGFR/HER2/HER3 and downstream effectors ERK and AKT in pSKBr3 and LRSKBr3 cells upon 24h lapatinib or veh treatment. (**b**) Western blot depicting the expression of AMPK and P-AMPK in pSKBr3 and LRSKBr3 cells upon 24h lapatinib or veh treatment. (**c**) Expression of ERRα, S6 and P-S6 in pSKBr3 and pSKBr3 (left) and LRSKBr3 (right) cells upon lapatinib or veh treatment in presence or absence of the mTORC1 inhibitor INK1341 treatment. Lap, lapatinib; veh, vehicle. (**d**) Western blot showing the expression of ERRα and of phosphorylated S6 (pS6) in SKBr3 cells that have been generated to become resistant to Trastuzumab treatment.

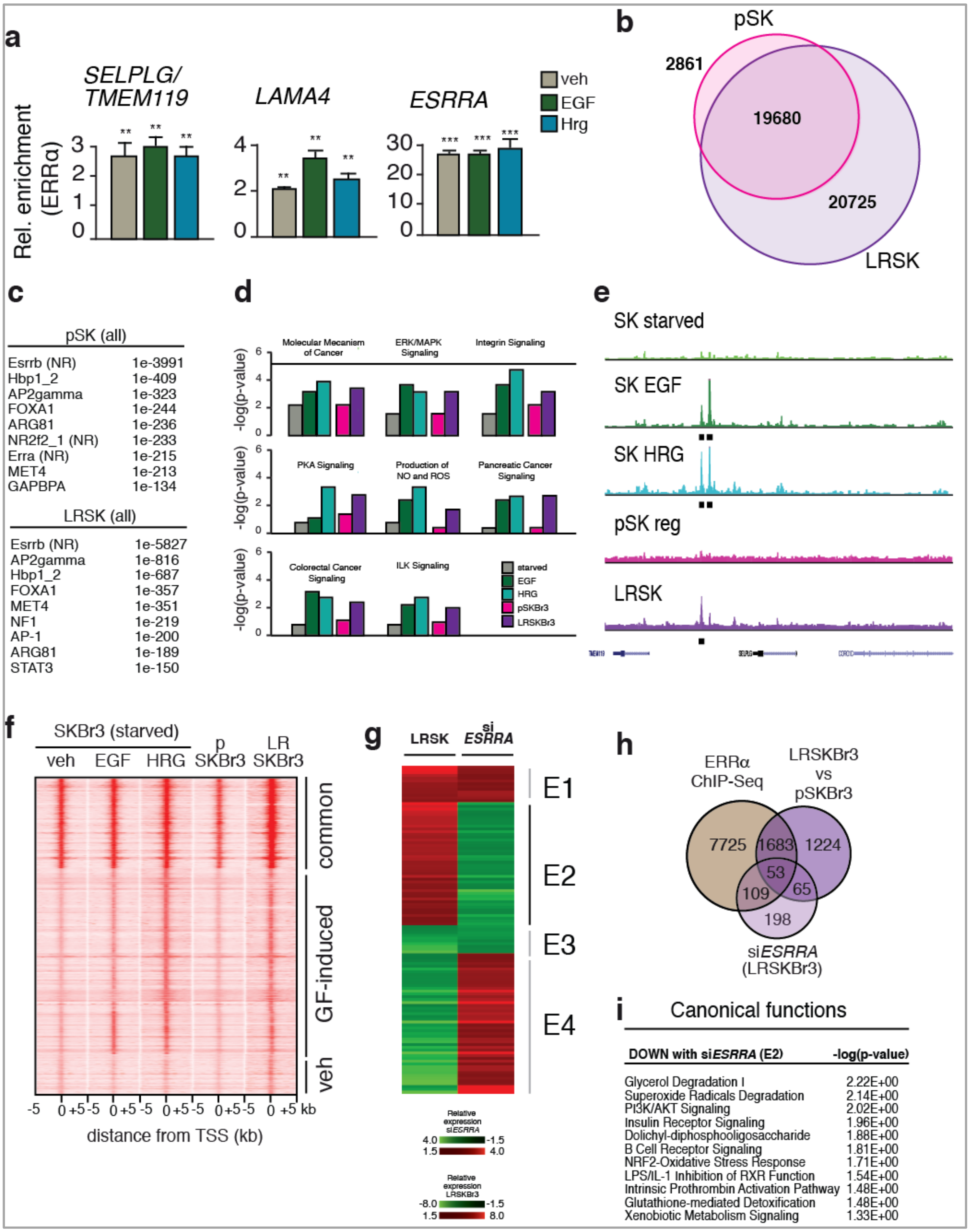
a
SELPLG/TMEM119
LAMA4
ESRRA
Rel. enrichment (ERRα)
veh
EGF
Hrg
b
pSK
LRSK
2861
19680
20725
c
pSK (all)
Esrrb (NR) 1e-3991
Hbp1_2 1e-409
AP2gamma 1e-323
FOXA1 1e-244
ARG81 1e-236
NR2f2_1 (NR) 1e-233
Erra (NR) 1e-215
MET4 1e-213
GAPBPA 1e-134
LRSK (all)
Esrrb (NR) 1e-5827
AP2gamma 1e-816
Hbp1_2 1e-687
FOXA1 1e-357
MET4 1e-351
NF1 1e-219
AP-1 1e-200
ARG81 1e-189
STAT3 1e-150
d
Molecular Mecanism of Cancer
ERK/MAPK Signaling
Integrin Signaling
PKA Signaling
Production of NO and ROS
Pancreatic Cancer Signaling
Colorectal Cancer Signaling
ILK Signaling
-log(p-value)
starved
EGF
HRG
pSKBr3
LRSKBr3
e
SK starved
SK EGF
SK HRG
pSK reg
LRSK
f
SKBr3 (starved)
veh
EGF
HRG
p SKBr3
LR SKBr3
common
GF-induced
veh
distance from TSS (kb)
g
LRSK
si ESRRA
E1
E2
E3
E4
Relative expression siESRRA
Relative expression LRSKBr3
h
ERRα ChIP-Seq
LRSKBr3 vs pSKBr3
siESRRA (LRSKBr3)
7725
1683
1224
53
109
65
198
i
Canonical functions
DOWN with siESRRA (E2) -log(p-value)
Glycerol Degradation I 2.22E+00
Superoxide Radicals Degradation 2.14E+00
PI3K/AKT Signaling 2.02E+00
Insulin Receptor Signaling 1.96E+00
Dolichyl-diphosphooligosaccharide 1.88E+00
B Cell Receptor Signaling 1.81E+00
NRF2-Oxidative Stress Response 1.71E+00
LPS/IL-1 Inhibition of RXR Function 1.54E+00
Intrinsic Prothrombin Activation Pathway 1.48E+00
Glutathione-mediated Detoxification 1.48E+00
Xenobiotic Metabolism Signaling 1.33E+00

Supplementary Figure 3 | ERRα transcriptional activity in lapatinib-resistant cells. (**a**) Standard ChIP experiment showing the enrichment of ERRα recruitment to growth factor-reprogrammed sites according to ChIP-Seq, upon EGF and HRG stimulation in LRSKBr3 cells maintained in serum-starved media. *ESRRA* is used as a non-reprogrammed bound segment. Representative graph of 3 independent experiments performed in triplicates, error bars represent standard error of the mean, statistical significance is calculated using 2-tailed unpaired t-test **, $p<0.01$; ***, $p<0.001$. (**b**) Venn diagram depicting the overlap observed upon ChIP-seq between genomic segments bound by ERRα in untreated SKBr3 and those bound in LRSKBr3 cells maintained in lapatinib. (**c**) Computational discovery of *de novo* motif binding sites in ERRα-bound sequences in untreated pSKBr3 cells and in LRSKBr3 cells maintained in lapatinib according to ChIP-Seq results. (**d**) Enrichment of canonical pathways by IPA associated with target genes bound by ERRα within +/-10kb of their TSS in pSKBr3 and LRSKBr3 cells. (**e**) Examples of binding profiles for ERRα on lapatinib resistant-reprogrammed sites in SKBr3 cells treated with veh, EGF or HRG and in pSKBr3 cells and LRSKBr3 cells maintained in lapatinib. (**f**) Heatmap showing the intensities of ERRα binding events in SKBr3 cells for each peak determined from ERRα ChIP-seq upon EGF or HRG and in pSKBr3 cells and LRSKBr3 cells maintained in lapatinib and clustered according to peaks commonly bound by ERRα of in growth factor induced binding sites. (**g**) Heatmap showing hierarchical clustering of the genes with expression profile significantly modulated in LRSKBr3 cells compared to pSKBR3 cells and compared to genes with expression significantly modulated in LRSKBr3 cells upon depletion of ERRα using siRNA. (**h**) Overlap between target genes with an ERRα-bound genomic segment at +/-10kb of their TSS, genes significantly modulated in LRSKBr3 cells compared to pSKBr3 cells and genes significantly modulated in LRSKBr3 cells upon depletion of ERRα using siRNA. (**i**) Enrichment of canonical pathways by IPA associated with common target genes from clusters defined in **(g)**.

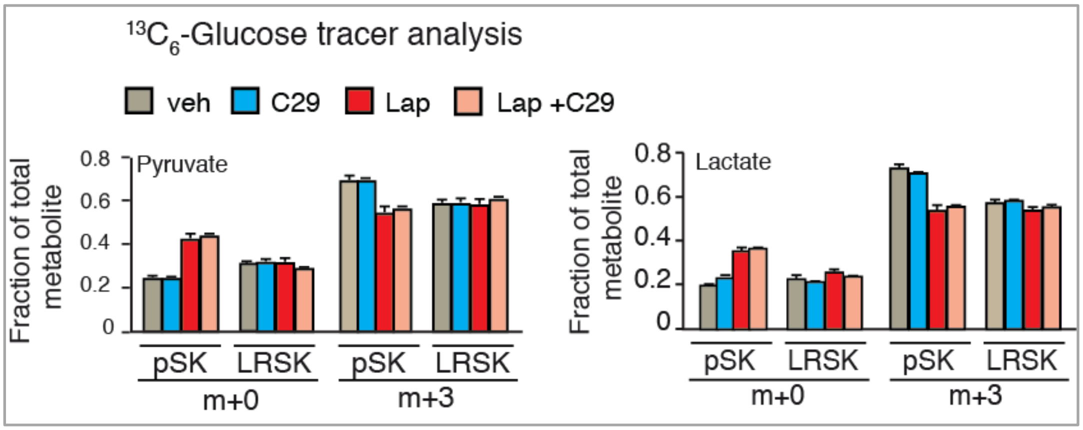

Supplementary Figure 4. $^{3}C_6$-glucose flux in parental and lapatinib-resistant SKBr3 cells. Quantification of the fraction of intermediate metabolites pyruvate (m+3), lactate (m+3) produced from the tracing uniformly $^{13}C_6$-labeled glucose in pSKBr3 and LRSKBr3 cells upon lapatinib treatment and pharmacological inhibition of ERRα with C29. The m+0 ion represents the fraction of un-labelled metabolite.

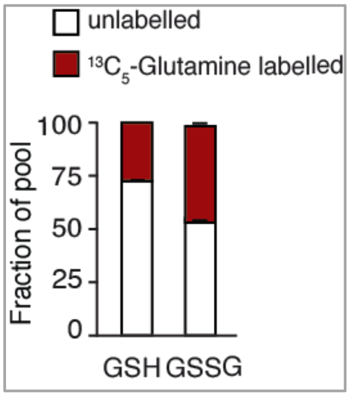

Supplementary Figure 5. Glutamine contribution to glutathione biosynthesis in SKBr3 cells. Fractional contribution of 13C5-glutamine into reduced glutathione (GSH) and oxidized glutathione (GSSG) biosynthesis in SKBr3 cells incubated for 6h in the presence of the tracer. Unlabelled fraction is m+0, labelled fraction is the fraction occupied by m+1 to m+8 isotopomers (GSH) or m+1 to m+12 isotopomers (GSSG). Data is shown as average values from biological triplicates taken from a representative experimental set.

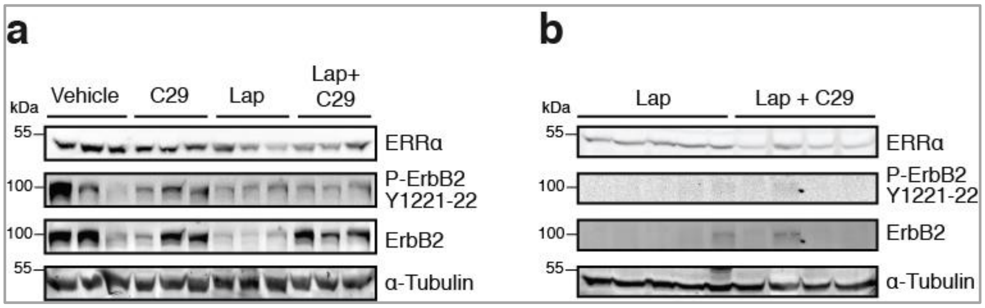

Supplementary Figure 6. ERBB2 and ERRα expression upon treatment with lapatinib and C29. (**a**) Western blot showing the quantification of ERRα, ErbB2 and P-ErbB2 Y1221-22 in NIC tumours from mice treated with vehicle, C29, Lapatinib or Lapatinib in combination with C29. α-tubulin is used as loading control. (**b**) Western blot showing the quantification of ERRα, ErbB2 and P-ErbB2 Y1221-22 in lapatinib-resistant NIC tumors from mice treated with vehicle, C29, Lapatinib or Lapatinib in combination with C29. α-tubulin is used as loading control.

Supplementary Table 1 | si*ESRRA* in SKBr3 (starved) with ERRα binding sites

**Canonical Pathway (DOWN)**	**-log (p-value)**	**GENES**
Role of IL-17F in Inflammatory Airway	1.95	TRAF3IP2,IGF1
Synaptic Long Term Depression	1.86	PNPLA8,PLA2G4E,IGF1
Phospholipases	1.75	PNPLA8,PLA2G4E
Eicosanoid Signalling	1.68	PNPLA8,PLA2G4E
MAPK Signalling in Influenza	1.62	PNPLA8,PLA2G4E
Growth Hormone Signalling	1.60	IGF1,CEBPA
Acyl-CoA Hydrolysis	1.38	ACOT7
Antioxidant Action of Vitamin C	1.34	PNPLA8,PLA2G4E
TCA Cycle II (Eukaryotic)	1.30	ACO2
**Canonical Pathway (UP)**	**-log (p-value)**	**GENES**
Small Cell Lung Cancer Signalling	1.87	FHIT,MAX
IL-4 Signalling	1.84	IL4R,SYNJ1
Melanocyte Development	1.72	ADCY5,ATF2
FGF Signalling	1.72	FGFR1,ATF2
GDP-glucose Biosynthesis	1.71	HK1
Glycogen Degradation II	1.61	AGL
Renin-Angiotensin Signalling	1.53	ADCY5,ATF2
PTEN Signalling	1.47	SYNJ1,FGFR1
p38 MAPK Signalling	1.46	MAX,ATF2
P2Y Purigenic Receptor Signalling	1.44	ADCY5,ATF2
PI3K Signalling in B Lymphocytes	1.40	IL4R,ATF2
GNRH Signalling	1.39	ADCY5,ATF2
D-myo-inositol (1,4,5)-trisphosphate	1.36	SYNJ1
Cardiomyocyte Differentiation	1.34	ATF2
Hepatic Fibrosis / Hepatic Stellate Cell	1.33	IL4R,FGFR1

Supplementary Table 2 | si*ESRRA* in SKBr3 (growth factors) with ERRα binding sites

Canonical Pathway (DOWN)	-log (p-value)	GENES
Glioblastoma Multiforme Signalling	2.71	WNT7A,WNT9A,SOS2,CDKN1A,NF2,PDGFRB
Fatty Acid Activation	2.25	ACSL6,ACSL4
Colanic Acid Building Blocks Biosynthesis	2.18	GALK2,UGP2
Î³-linolenate Biosynthesis II (Animals)	2.02	ACSL6,ACSL4
Mitochondrial L-carnitine Shuttle Pathway	2.02	ACSL6,ACSL4
Putrescine Degradation III	2.02	IL4I1,ALDH1B1
LPS/IL-1 Mediated Inhibition of RXR	1.87	PPARA,IL4I1,ALDH1B1,ACSL6,CHST11,ACSL4
Role of NANOG in ES Cell Pluripotency	1.78	WNT7A,WNT9A,SOS2,BMP8
Flavin Biosynthesis IV (Mammalian)	1.76	RFK
Wnt/Î²-catenin Signalling	1.75	WNT7A,WNT9A,RARB,KREMEN1,RARG
ERK5 Signalling	1.72	CREB1,GNA13,ELK4
JAK/Stat Signalling		
Regulation of the EMT Pathway	1.66	WNT7A,WNT9A,SOS2,PDGFRB,PSEN1
Basal Cell Carcinoma Signalling	1.59	WNT7A,WNT9A,BMP8B
Thyroid Hormone Biosynthesis in	1.58	IYD
BMP signalling	1.54	CREB1,PITX2,BMP8B

pathway		
Fatty Acid b-oxidation I	1.54	ACSL6,ACSL4
Human Embryonic Stem Cell Pluripotency	1.51	WNT7A,WNT9A,BMP8B,PDGFRB
Aryl Hydrocarbon Receptor Signalling	1.45	ALDH1B1,CDKN1A,RARB,RARG
Axonal Guidance Signalling	1.44	MYL9,SRGAP3,WNT7A,WNT9A SOS2,GNA13,BMP8B,EIF4E
Prostate Cancer Signalling	1.43	SOS2,CREB1,CDKN1A
Stearate Biosynthesis I (Animals)	1.42	ACSL6,ACSL4
PAK Signalling	1.37	MYL9,SOS2,PDGFRB
Lysine Degradation II	1.36	AADAT
Galactose Degradation I (Leloir Pathway)	1.36	GALK2
Cardiac Hypertrophy Signalling	1.36	MYL9,CACNA1E,CREB1,GNA13,EIF4E
Role of THOP1 in Alzheimer's Disease	1.34	KNG1,CREB1
PPAR Signalling	1.34	PPARA,SOS2,PDGFRB
SAPK/JNK Signalling	1.32	MAP4K3,SOS2,GNA13
Glycogen Biosynthesis II (UDP-D-Glu)	1.32	UGP2
IGF-1 Signalling	1.32	SOS2,SOCS4,IGFBP1
Molecular Mechanisms of Cancer	1.31	SOS2,CDKN1A,GNA13,BMP8B,RALGDS,PSEN1
Phospholipases	1.31	PLD3,PLA2G2C
PI3K/AKT Signalling	1.30	SOS2,CDKN1A,EIF4E

**Canonical Pathway (UP)**	**-log(p-**	**GENES**

	**value)**	
Ephrin Receptor Signalling	1.78	MAP3K14,RGS3,GNAI1,ABL1,PGF
IL-10 Signalling	1.70	SOCS3,MAP3K14,CD14
Intrinsic Prothrombin Activation	1.63	COL1A2,COL11A2
Axonal Guidance Signalling	1.53	SRGAP3,BMP4,RGS3,UNC5B,GNAI1,ABL1,BMP8B,PGF
Biotin-carboxyl Carrier Protein	1.48	HLCS
Lactose Degradation III	1.48	LCT
Factors Promoting Cardiogenesis	1.41	BMP4,DKK1,BMP8B
Bladder Cancer Signalling	1.40	FGF21,ABL1,PGF

Supplementary Table 3 | LRSKBr3 vs pSK (canonical pathways)

UP	-log (p-value)	DOWN	-log (p-value)
Cell Cycle Control of Chromosomal Replication	1.26E+01	D-myo-inositol (1,4,5)-Trisphosphate Biosynthesis	2.38E+00
Role of BRCA1 in DNA Damage Response	1.16E+01	NRF2-mediated Oxidative Stress Response	2.19E+00
Role of CHK Proteins in Cell Cycle Checkpoint Control	1.10E+01	Aldosterone Signalling in Epithelial Cells	1.97E+00
Mismatch Repair in Eukaryotes	9.21E+00	Thiamin Salvage III	1.92E+00
Hereditary Breast Cancer Signalling	8.21E+00	Mechanisms of Viral Exit from Host Cells	1.90E+00
Estrogen-mediated S-phase Entry	7.52E+00	Methylglyoxal Degradation III	1.80E+00
ATM Signalling	7.13E+00	Glutathione Redox Reactions I	1.75E+00
Mitotic Roles of Polo-Like Kinase	6.61E+00	Role of NFAT in Cardiac Hypertrophy	1.62E+00
Cell Cycle: G2/M DNA Damage Checkpoint Regulation	6.36E+00	Glutamine Biosynthesis I	1.62E+00
Aryl Hydrocarbon Receptor Signalling	5.52E+00	Phospholipase C Signalling	1.61E+00
Cell Cycle: G1/S Checkpoint Regulation	5.06E+00	Gap Junction Signalling	1.38E+00
Cyclins and Cell Cycle Regulation	4.96E+00	Glutathione-mediated Detoxification	1.35E+00
GADD45 Signalling	4.20E+00	Actin Cytoskeleton Signalling	1.33E+00
Breast Cancer Regulation by Stathmin1	3.95E+00	Heme Biosynthesis from Uroporphyrinogen-III I	1.32E+00
Gap Junction Signalling	3.70E+00	NAD Biosynthesis III	1.32E+00
Molecular Mechanisms of Cancer	3.61E+00	Corticotropin Releasing Hormone Signalling	1.31E+00
14-3-3-mediated Signalling	3.54E+00	Agrin Interactions at Neuromuscular Junction	1.30E+00
Remodelling of Epithelial Adherens Junctions	3.11E+00		
p53 Signalling	2.96E+00		
Sertoli Cell-Sertoli Cell Junction Signalling	2.68E+00		
Antiproliferative Role of TOB in T Cell Signalling	2.61E+00		
Prostate Cancer Signalling	2.60E+00		

Salvage Pathways of Pyrimidine Ribonucleotides	2.54E+00		
Pyridoxal 5'-phosphate Salvage Pathway	2.49E+00		
Pyrimidine Deoxyribonucleotides De Novo Biosyn	2.48E+00		
Small Cell Lung Cancer Signalling	2.30E+00		
Epithelial Adherens Junction Signalling	2.26E+00		
RAN Signalling	2.23E+00		
Interferon Signalling	2.18E+00		
Superoxide Radicals Degradation	2.16E+00		
DNA damage-induced 14-3-3Ïƒ Signalling	2.09E+00		
Regulation of Cellular Mechanics by Calpain Protease	2.06E+00		
Antigen Presentation Pathway	2.05E+00		
Ovarian Cancer Signalling	2.00E +00		
Pancreatic Adenocarcinoma Signalling	2.00E+00		
Pyrimidine Ribonucleotides Interconversion	1.91E+00		
Hepatic Cholestasis	1.90E+00		
Pyrimidine Ribonucleotides De Novo Biosynthesis	1.80E+00		
Hypoxia Signalling in the Cardiovascular System	1.80E+00		
Role of MAPK Signalling	1.78E+00		
IL-17A Signalling in Gastric Cells	1.76E+00		
Thio-molybdenum Cofactor Biosynthesis	1.65E+00		
Xanthine and Xanthosine Salvage	1.65E+00		
Glutathione-mediated Detoxification	1.62E+00		
cAMP-mediated signalling	1.58E+00		
LPS/IL-1 Mediated Inhibition of RXR Function	1.57E+00		
Ubiquinol-10 Biosynthesis (Eukaryotic)	1.55E+00		

Protein Ubiquitination Pathway	1.55E+00			
Toll-like Receptor Signalling	1.44E+00			
Triacylglycerol Biosynthesis	1.44E+00			
IL-17A Signalling in Fibroblasts	1.37E+00			
CDP-diacylglycerol Biosynthesis I	1.37E+00			
Methionine Degradation I (to Homocysteine)	1.37E+00			
Guanine and Guanosine Salvage I	1.36E+00			
Adenine and Adenosine Salvage I	1.36E+00			
Putrescine Biosynthesis III	1.36E+00			
Colorectal Cancer Metastasis Signalling	1.35E+00			
Lysine Degradation II	9.72E-01			
Cysteine Biosynthesis III (mammalia)	1.27E+00			
Antioxidant Action of Vitamin C	1.20E+00			
NRF2-mediated Oxidative Stress Response	9.66E-01			
Glucose and Glucose-1-phosphate Degradation	7.36E-01			
Glutathione-mediated Detoxification	1.62E+00			
Xenobiotic Metabolism Signalling	5.09E-01			
Glycogen Degradation II	6.95E-01			
Ketolysis	6.95E-01			
Ketogenesis	6.58E-01			

Supplementary Table 4 | si*ESRRA* in LRSKBr3 (canonical pathways)

DOWN	-log (p-value)	UP	-log (p-value)
Telomere Extension by Telomerase	2.04E+00	non significant	
L-cysteine Degradation II	2.01E+00		
Cysteine Biosynthesis/Homocysteine	1.71E+00		
Role of Tissue Factor in Cancer	1.63E+00		
CD40 Signalling	1.63E+00		
Growth Hormone Signalling	1.53E+00		
Phenylethylamine Degradation I	1.42E+00		
Coagulation System	1.40E+00		
Glycerol Degradation I	1.39E+00		
Lysine Degradation II	1.38E+00		
Antigen Presentation Pathway	1.37E+00		
Inhibition of Matrix Metalloprotease	1.36E+00		
Bladder Cancer Signalling	1.35E+00		
Acetyl-CoA Biosynthesis I (PDH)	1.34E+00		
Superoxide Radicals Degradation	1.34E+00		
Glycogen Biosynthesis II	1.33E+00		
Ketolysis	1.31E+00		
Ketogenesis	1.30E+00		

Supplementary Table 5 | siESRRA in LRSKBr3 _AND_LRSKBr3 vs pSK (pathways)

DOWN	-log(p-value)	UP	-log(p-value)
LPS/IL-1 Mediated Inhibition of RXR Function	2.04E+00	Methionine Degradation I (to Homocysteine)	1.45E+00
Glycerol Degradation I	2.01E+00	Extrinsic Prothrombin Activation Pathway	1.42E+00
Superoxide Radicals Degradation	1.93E+00	Leukotriene Biosynthesis	1.42E+00
FGF Signalling	1.91E+00	Cysteine Biosynthesis III (mammalia)	1.39E+00
Xenobiotic Metabolism Signalling	1.74E+00		
Role of Tissue Factor in Cancer	1.70E+00		
Dolichyl-diphosphooligosaccharide Biosynthesis	1.67E+00		
PI3K/AKT Signalling	1.62E+00		
Insulin Receptor Signalling	1.56E+00		
Extrinsic Prothrombin Activation Pathway	1.51E+00		
B Cell Receptor Signalling	1.41E+00		
NRF2-mediated Oxidative Stress Response	1.36E+00		
Regulation of the EMT	1.35E+00		
Clathrin-mediated Endocytosis Signalling	1.33E+00		
Intrinsic Prothrombin Activation Pathway	1.33E+00		
Glutathione-mediated Detoxification	1.32E+00		
Dermatan Sulfate Biosynthesis (Late Stages)	1.30E+00		

Supplementary Table 6 | LRSKBr3 vs pSK with ERRα binding site (ChIP-Seq) (pathways)

DOWN	-log (p-value)	UP	-log (p-value)
Mechanisms of Viral Exit from Host Cells	5.59E+00	Estrogen-mediated S-phase Entry	7.37E+00
NRF2-mediated Oxidative Stress Response	5.57E+00	Role of CHK Proteins in Cell Cycle Checkpoint	6.06E+00
Xenobiotic Metabolism Signalling	3.70E+00	Aryl Hydrocarbon Receptor Signalling	5.79E+00
nNOS Signalling in Neurons	3.33E+00	Mitotic Roles of Polo-Like Kinase	5.78E+00
Amyloid Processing	3.09E+00	Cell Cycle Regulation by BTG Family Proteins	5.60E+00
Leukocyte Extravasation Signalling	3.04E+00	Role of BRCA1 in DNA Damage Response	5.60E+00
p70S6K Signalling	3.03E+00	ATM Signalling	5.60E+00
Regulation of Cellular Mech by Calpain	2.76E+00	Mismatch Repair in Eukaryotes	5.48E+00
Sertoli Cell-Sertoli Cell Junction Signalling	2.74E+00	Cyclins and Cell Cycle Regulation	4.83E+00
Tight Junction Signalling	2.51E+00	Protein Ubiquitination Pathway	4.75E+00
Germ Cell-Sertoli Cell Junction Signalling	2.44E+00	Cell Cycle: G2/M DNA Damage Checkpoint Reg	4.63E+00
Glutathione Redox Reactions I	2.42E+00	Hypoxia Signalling in the Cardiovascular System	4.40E+00
Mitochondrial Dysfunction	2.08E+00	PI3K/AKT Signalling	4.33E+00
Phenylethylamine Degradation I	2.05E+00	Hereditary Breast Cancer Signalling	4.10E+00
Myo-inositol Biosynthesis	2.05E+00	Cell Cycle: G1/S Checkpoint Regulation	3.98E+00
ErbB4 Signalling	2.03E+00	ERK5 Signalling	3.85E+00
PI3K/AKT Signalling	2.01E+00	Cell Cycle Control of Chromosomal Replication	3.67E+00
Aryl Hydrocarbon Receptor Signalling	2.01E+00	Molecular Mechanisms of Cancer	3.63E+00
Breast Cancer Regulation by Stathmin1	2.00E+00	Remodeling of Epithelial Adherens Junctions	2.99E+00
HER-2 Signalling in Breast Cancer	1.99E+00	Purine Nucleotides Degradation II (Aerobic)	2.98E+00
VEGF Signalling	1.97E+00	Pancreatic Adenocarcinoma Signalling	2.91E+00
Calcium-induced T	1.96E+00	Purine Nucleotides De Novo	2.79E+00

Lymphocyte Apoptosis		Biosynthesis II	
Integrin Signalling	1.92E+00	Salvage Pathways of Pyrimidine Ribo	2.77E+00
PI3K Signalling in B Lymphocytes	1.89E+00	Adenine and Adenosine Salvage III	2.76E+00
ERK5 Signalling	1.88E+00	GADD45 Signalling	2.76E+00
Histamine Degradation	1.88E+00	Guanine and Guanosine Salvage I	2.68E+00
Epithelial Adherens Junction Signalling	1.87E+00	Urate Biosynthesis/Inosine 5'-phosphate	2.65E+00
Pentose Phosphate Pathway (Oxidative)	1.84E+00	Pyridoxal 5'-phosphate Salvage Pathway	2.56E+00
Triacylglycerol Degradation	1.80E+00	NRF2-mediated Oxidative Stress Response	2.41E+00
Phenylalanine Degradation IV (Mammalian,)	1.79E+00	Adenosine Nucleotides Degradation II	2.40E+00
Aldosterone Signalling in Epithelial Cells	1.75E+00	Aldosterone Signalling in Epithelial Cells	2.37E+00
Telomerase Signalling	1.73E+00	Prostate Cancer Signalling	2.34E+00
Chemokine Signalling	1.72E+00	IL-17A Signalling in Fibroblasts	2.32E+00
CDP-diacylglycerol Biosynthesis I	1.71E+00	IL-17A Signalling in Gastric Cells	2.30E+00
Methionine Degradation I (to Homocysteine)	1.71E+00	Granzyme B Signalling	2.29E+00
mTOR Signalling	1.70E+00	Androgen Signalling	2.28E+00
IL-15 Production	1.68E+00	Inosine-5'-phosphate Biosynthesis II	2.21E+00
Superoxide Radicals Degradation	1.68E+00	Huntington's Disease Signalling	2.15E+00
Gap Junction Signalling	1.67E+00	Assembly of RNA Polymerase II Complex	2.13E+00
Factors Promoting Cardiogenesis in Vertebrates	1.63E+00	Breast Cancer Regulation by Stathmin1	2.11E+00
Apoptosis Signalling	1.63E+00	CD27 Signalling in Lymphocytes	2.08E+00
Glutathione-mediated Detoxification	1.63E+00	Guanosine Nucleotides Degradation III	1.81E+00
Oxidative Ethanol Degradation III	1.63E+00	Small Cell Lung Cancer Signalling	1.80E+00
UVC-Induced MAPK Signalling	1.61E+00	Colorectal Cancer Metastasis Signalling	1.79E+00
Phosphatidylglycerol Biosynthesis II	1.56E+00	TWEAK Signalling	1.79E+00
Putrescine Degradation III	1.56E+00	GNRH Signalling	1.78E+00

Cysteine Biosynthesis III (mammalia)	1.56E+00	14-3-3-mediated Signalling	1.73E+00
Thiamin Salvage III	1.40E+00	PTEN Signalling	1.73E+00
Methylglyoxal Degradation VI	1.40E+00	Acetate Conversion to Acetyl-CoA	1.72E+00
Triacylglycerol Biosynthesis	1.40E+00	HIF1Î± Signalling	1.70E+00
Cholecystokinin/Gastrin-mediated Signalling	1.33E+00	Xenobiotic Metabolism Signalling	1.62E+00
Paxillin Signalling	1.33E+00	ILK Signalling	1.59E+00
Stearate Biosynthesis I (Animals)	1.32E+00	Superoxide Radicals Degradation	1.55E+00
		Methionine Degradation I (to Homocysteine)	1.54E+00
		Glutathione-mediated Detoxification	1.43E+00
		Purine Ribonucleosides Degradation	1.42E+00
		Cysteine Biosynthesis III (mammalia)	1.39E+00
		Xanthine and Xanthosine Salvage	1.34E+00

Supplementary Table 7 | si*ESRRA* in LRSKBr3 with ERRα binding site (ChIP-Seq) (pathways)

DOWN	-log(p-value)	UP	-log(p-value)
L-cysteine Degradation II	2.36E+00	Signalling	1.69E+00
Cysteine Biosynthesis/Homocysteine	2.06E+00	Phosphatidylcholine Biosynthesis I	1.62E+00
Role of Tissue Factor in Cancer	1.89E+00	Sphingosine and Sphingosine-1-phosphate	1.56E+00
Insulin Receptor Signalling	1.70E+00	Hematopoiesis from Multipotent Stem Cells	1.39E+00
Glycerol Degradation I	1.66E+00	Choline Biosynthesis III	1.36E+00
Acetyl-CoA Biosynthesis I (PDH)	1.59E+00	Methionine Degradation I (to Homocysteine)	1.30E+00
Superoxide Radicals Degradation	1.59E+00		
Glycogen Biosynthesis II	1.59E+00		
Death Receptor Signalling	1.57E+00		
Aldosterone Signalling in Epithelial Cells	1.54E+00		
CD40 Signalling	1.51E+00		
ERK5 Signalling	1.50E+00		
B Cell Receptor Signalling	1.49E+00		
Sphingomyelin Metabolism	1.46E+00		
GDNF Family Ligand-Receptor	1.45E+00		
Renal Cell Carcinoma Signalling	1.42E+00		
Ketolysis	1.37E+00		
Ketogenesis	1.33E+00		
Dolichyl-diphosphooligosaccharide	1.33E+00		
Ceramide Signalling	1.32E+00		
Cysteine Biosynthesis III	1.30E+00		
TCA Cycle II (Eukaryotic)	1.30E+00		

Supplementary Table 8 | ChIP Primers

Name	Sequence
EGF-1-U	TGGTGATGAGTCATCTGAATGCAAGC
EGF-1L	GGTGAACTTTGGGACTCAGATTTTGG
EGF-2-U	CACACAGACATACAAGCCAACAAGTG
EGF-2L	TGTCTGCTTGTCTATCCATTTACCTG
EGF-3-U	CGAGAACATTGTAGACTACCTGCC
EGF-3L	TCTCTCTTCCCACTATTCTGAGCC
PARVB-U	AGATGGGCAAACACAGGCTTAGAG
PARVB-L	TTAAATGCCAGGAATGACATCAGC
miR-449B-3P-U	ACTCTCTGTGTGACCTTGGGCACG
miR-449B-3P-L	CCACCAGGCACAAAGGTAACAAGA
TMEM119-SELPLG-U	CATCATGTCAAGGGCATTTTTCCA
TMEM119-SELPLG-L	CCCTCAGCCTCTAGTGTGATCCTA
CD86-U	CTTGACCTTTGCAGAACACTCCCA
CD86-L	CCAGATTATTTCCACCGACAGCAG
LAMA4-U	GCATTCAGAAACGCTTCTTGCTCA
LAMA4-L	TGCACTAGTCACTGGTGACACAGC
EGF-9-U	ATTCCCAGGCACTCAGAGCATGGC
EGF-9L	GGCAAGGTCCGCACTGAGTCACAG
EGF-10-U	CTCCTTGGAAGCAGAGAGTGACCG
EGF-10L	GGTCAGGTCAAACCACAGCTTCCT
EGF-11-U	CCTCAGGCTCAGTCCATTGACTCT
EGF-11L	CCACAGCTTCCTCTGACTCAATGT
EGF-12-U	CAGCATGGTTTGGTGATTCATTGA
EGF-12L	CGTGTGTGTTATTTCCAGGGTGAG
EGF-13-U	CAGTCATGTGGAAGGGCAGGAGAG
EGF-13L	CTGATCTCAGCCTCCCACCTCCAC
EGF-14-U	GCGTGACTCACAATCTTTCCCACT
EGF-14L	GCAGCAGACAGAGCAGTGACAGTC
EGF-15-U	CCTGCCAGTGACCCCACCTTCCAG
EGF-15L	GCCACAGCAACCCACAGAGAAGTG
EGF-16-U	CCACTTTGTGCCAGTCTCCTGTAC
EGF-16L	GTGACTCAGGTGCCCTTCTCTGTC
EGF-17-U	GCATTCATTCCAAATCAGCACACG
EGF-17L	CCTCAGGGCTGAGCACTGTATTAA
h18S-U	GATGGGCGGCGGAAAATAG
h18S-L	GCGTGGATTCTGCATAATGGT
ERRneg-U	gtggcccacaggtgtcgctcaagtcttc
ERRneg-L	ggatgcagtgtccttctcccccagattg
hGSR-F	TGATTCAATGATCAGCACCAAC

hGSR-R	CAGTAACCATGCTGACTTCCA
hSOD3-F	AGAGAAAGCTCTCTTGGAGGA
hSOD3-R	GGCGTACATGTCTCGGAT
hSOD1-F	GGCATCATCAATTTCGAGCA
hSOD1-R	GGCCTTCAGTCAGTCCTTT
hGPX1 F	CGGGGCAAGGTACTACTTAT
hGPX1 R	GTTCTTGGCGTTCTCCTGAT
hSOD2 F	GCTGCTCTATTGTAGCATTTCT
hSOD2 R	CATCCCTACAAGTCCCCAAA

Supplementary Table 9 | Specific metabolite transitions for quantifier/qualifier ions and ESI modes for GC/MS

Metabolite	Molecular formula	m/z	RT (min)
Pyruvate (methoxime, TBDMS)	C6H12NO3Si	171-177 (115)	8.926
Lactate (di-TBDMS)	C11H25 O3Si	261-264 (233)	11.73
Succinate (di-TBDMS)	C12H25 O3Si2	289-292 (331)	14.845
Fumarate (di-TBDMS	C12H23O4Si2	287-290 (329)	15.221
Alpha-Ketoglutarate (methoxime, di-TBDMS)	C14H28NO5Si2	346-350 (258)	17.331
Malate (tri-TBDMS)	C18H39O5Si3	419-422 (287)	18.239
Glutamate (di-TBDMS)	C19H42NO4Si3	432-437 (330)	19.709
Citrate (tetra-TBDMS)	C20H39O6Si3	459-464 (431,403)	22.394
Isocitrate (tetra-TBDMS)	C20H39O6Si3	459-464 (431,403)	22.477
Myristic acid-D27, used as an internal standard, was monitored at m/z 312 (17.979 min). Qualifying ions are indicated in brackets to the right of the quantifying ions			

Supplementary Table 10 | Specific metabolite transitions for quantifier/qualifier ions and ESI modes for LC/MS

Compound	Precursor ion (m/z)	Quantifier ion (m/z)	Collision energy (V)	Qualifier ion (m/z)	Collision energy (V)
GSH (M0-M+8)*	308 (308-316)	179	4	76.1	28
GSSG (M0-M+12)*	613 (613-625)	355.1	16	231.1	28
Gln	147	130	4	84.1	16
Glu	148	130.1	4	84.1	12
Gly	76	47.2	16	42.1	48
Cys	122	76.1	12	59.1	24
γ-Glu-Cys	251	122.1	4	84.1	24
Hyp	132	68.1	20	86.1	20
αKG	145	101	0	57.1	4
Compounds from the Sha method are detected in positive mode and αKG (Shn method) is negative mode.					
Hyp = Hydroxyproline.					
*range of m/z values detected in labeling experiments					

Supplementary Methods

GC/MS and mass isotopomer distribution analysis. Cells were treated and pulsed with 10 mM U-^{13}C-glucose (uniformly labelled, Cambridge Isotope Laboratories, CLM-1396, 99%) or 2 mM U-^{13}C-glutamine (Cambridge Isotope Laboratories, CLM-1822, 97-99%) as described in Experimental Procedures. After 30 minutes, media was removed and plates were put on ice. Cells were washed with 2 ml of ice cold saline solution (9g/l NaCl) and quenched with 600 µl 80% methanol (v/v) on dry ice. Cells were harvested and sonicated 10 min at a high setting (30 sec on, 30 sec off) with a Biorupter bath sonicator (Diagenode). Lysates were cleared at 21,000 g for 10 min at 4ºC. Supernatants were transferred to cold vacuum centrifuge (Labconco) at -4ºC and evaporated to complete dryness. Pellets were dissolved in 30 µl pyridine containing 10 mg/ml methoxyamine hydrochloride (Sigma), sonicated, vortexed, and heated at 70ºC for 30 min before centrifugation. Supernatant were transferred to GC/MS auto-injection amber vials. Following addition of 70 µL of *N*-*tert*-Butyldimethylsilyl-*N*-methyltrifluoroacetamide (MTBSTA, 394882, Sigma)), samples were heated for an additional hour at 70ºC. GC/MS instrumentation and software (Agilent) were the same as previously described [2]. One µl of derivatised sample was injected into GC/MS. Acquisition methods were performed using retention time locking on Myristic acid-D_{27} (RT : 17.98 min). After retention time and spectral confirmation against authentic standards, data acquisition for stable isotope tracer analysis was done with Single Ion Monitoring (SIM). The fragments analysed are presented in Supplementary Table 10. Fragment identification required acceptable retention time, mass spectrum (acquired in scan mode), the presence of all quantifying ions (M+K, where M is the atomic mass of the M-57 fragment of TBDMS-

derivatised metabolites, and K the number of possible ^{13}C) in SIM acquisition, and the presence of at least 1 qualifying ion. Ion integrals were corrected for the contribution of naturally occurring isotopes (^{2}H, ^{3}H, ^{13}C, ^{14}C, ^{15}N, etc.) using fragment-specific matrices as described previously published in-house algorithm [3]. The final data represented the proportional flux where each m+i (with i : 0→K) represents a fraction of the total pool of metabolite extracted from the sample, the latter being set at 1. The use of a thirty minute pulse time prevents multiple iterations of the citric acid cycle, to allowing the analysis of early and unsaturated isotopic enrichments.

LC/MS Metabolite measurements. Metabolite measurements: The authentic metabolite standards were purchased from Sigma-Aldrich Co., while the following LC/MS grade solvents and additives were purchased from Fisher: ammonium acetate, formic acid, water, methanol, and acetonitrile. Sample extraction: Metabolites from pSKBr3 and LRSKBr3 cells ($1.5x10^{7}$ cells) were extracted using 380 µl of LC/MS grade 50% methanol/50% water mixture and 220 µl of cold acetonitrile. Samples were then homogenized by bead beating 2 min at 30 Hz (TissueLyser, Qiagen). A volume of 300 ul of ice-cold water and 600 ul of ice-cold methylene chloride were added to the lysates. Samples were vortexed and allowed to rest on ice for 10 min for phase separation followed by centrifugation at 4,000 rpm for 5 min. The upper aqueous layer was transferred to a fresh pre-chilled tube. Samples were dried by vacuum centrifugation operating at -4°C (Labconco). Samples were re-suspended in 50 µl of water and clarified by centrifugation for 15 min at 15,000 rpm at 1°C. Samples were maintained at 4°C for the duration of the LC-MS/MS analysis in the autosampler. LC-MS/MS analysis:

Samples were separated by U-HPLC (Ultra-High Performance Liquid Chromatography) (1290 Infinity, Agilent Technologies) using a Scherzo SM-C18 (3 mm x 150 mm) 3 µm column and guard column (Imtakt USA) operating at 10°C. For LC/MS analysis using method 1, solvent "A" consisted of 5 mM ammonium acetate in water and solvent "B" consisted of 200 mM ammonium acetate in 80% water and 20% acetonitrile. Metabolites were separated using a linear gradient from 0% to 100% "B" over a period of 5 min followed by 5 min at 100% "B" at flow rate of 0.4 ml/min. For LC/MS analysis using method 2, solvent "A" consisted of 100 mM formic acid in water and solvent "B" consisted of 200 mM ammonium formate in 30% acetonitrile and 70% water (adjusted to pH 8 using NH_4OH). Metabolites were separated using 100% A (0-2 min), then a linear gradient from 0% to 80% "B" (2-8 min) followed by 5 min at 100% "B" (8-13 min). For LC/MS analysis using method 3, solvent "A" consisted of 0.2% formic acid in water and solvent "B" consisted of 0.2% formic acid in MeOH. Metabolites were separated using 100% A (0-2 min), then a linear gradient from 0% to 80% "B" (2-8 min) followed by 5 min at 100% "B" (8-13 min). Flow rate was 0.4 mL/min for methods 1, 2 and 3. For all of the methods used above, the column was re-equilibrated for 6 min at 100% "A" before the next run. Metabolites were eluted into an electrospray ionization source (ESI) and detected by Multiple Reaction Monitoring (MRM) using a triple quadrupole mass spectrometer (6430 QQQ, Agilent Technologies). Specific metabolite transitions for quantifier/qualifier ions and ESI modes are listed in Supplemental Table S10. Quantifying ion integrated intensities were compared to external calibration curves collected at the same time as sample mass spectrometric data acquisition. The table S10

includes LC run conditions as well as ESI mode and MRM transitions chosen for absolute quantification.

For glutathione fractional labelling assessment, the procedure was modified as follow. Cells were pre-equilibrated in regular media for 2h, media was then replaced for media containing 4 mM 13C5-glutamine (Sigma-Aldrich, 605166, 98% atom) for 6h. Cells were washed twice with cold 150 mM ammonium formate pH 7.2 and quenched with 600 µl acetonitrile/methanol/water (3:5:2) extraction buffer on dry ice. Three ceramic beads were added to each tubes and samples were vortexed 30 sec and sonicated 5 min at a high setting (30 sec on, 30 sec off) with a Biorupter bath sonicator (Diagenode) in a cold room. 600 µL dichloromethane and 300 µL water were added to each samples and tubes were vigorously shaken for 15 sec. Samples were left undisturbed on ice for 10 min then lysates were cleared at 8,000 g for 5 min at 4°C. Upper aqueous phases were transferred to cold centri-trap (Labconco) at -4°C and evaporated to complete dryness.

References

1. Possemato R, *et al.* Functional genomics reveal that the serine synthesis pathway is essential in breast cancer. *Nature* **476**, 346-350 (2011).

2. Morita M, *et al.* mTORC1 controls mitochondrial activity and biogenesis through 4E-BP-dependent translational regulation. *Cell Metab* **18**, 698-711 (2013).

3. Nanchen A, Fuhrer T, Sauer U. Determination of metabolic flux ratios from 13C-experiments and gas chromatography-mass spectrometry data: protocol and principles. *Methods Mol Biol* **358**, 177-197 (2007).
